# Distinctive prosodic features of people with autism spectrum disorder: a systematic review and meta-analysis study

**DOI:** 10.1038/s41598-021-02487-6

**Published:** 2021-11-29

**Authors:** Seyedeh Zahra Asghari, Sajjad Farashi, Saeid Bashirian, Ensiyeh Jenabi

**Affiliations:** 1 School of Medicine, Hamadan University of Medical Sciences, Hamadan, Iran; 2 Autism Spectrum Disorders Research Center, Hamadan University of Medical Sciences, Hamadan, Iran; 3 Department of Public Health, School of Health, Hamadan University of Medical Sciences, Hamadan, Iran

**Keywords:** Diagnostic markers, Predictive markers, Paediatric research

## Abstract

In this systematic review, we analyzed and evaluated the findings of studies on prosodic features of vocal productions of people with autism spectrum disorder (ASD) in order to recognize the statistically significant, most confirmed and reliable prosodic differences distinguishing people with ASD from typically developing individuals. Using suitable keywords, three major databases including Web of Science, PubMed and Scopus, were searched. The results for prosodic features such as mean pitch, pitch range and variability, speech rate, intensity and voice duration were extracted from eligible studies. The pooled standard mean difference between ASD and control groups was extracted or calculated. Using I^2^ statistic and Cochrane Q-test, between-study heterogeneity was evaluated. Furthermore, publication bias was assessed using funnel plot and its significance was evaluated using Egger’s and Begg’s tests. Thirty-nine eligible studies were retrieved (including 910 and 850 participants for ASD and control groups, respectively). This systematic review and meta-analysis showed that ASD group members had a significantly larger mean pitch (SMD =  − 0.4, 95% CI [− 0.70, − 0.10]), larger pitch range (SMD =  − 0.78, 95% CI [− 1.34, − 0.21]), longer voice duration (SMD =  − 0.43, 95% CI [− 0.72, − 0.15]), and larger pitch variability (SMD = − 0.46, 95% CI [− 0.84, − 0.08]), compared with typically developing control group. However, no significant differences in pitch standard deviation, voice intensity and speech rate were found between groups. Chronological age of participants and voice elicitation tasks were two sources of between-study heterogeneity. Furthermore, no publication bias was observed during analyses (p > 0.05). Mean pitch, pitch range, pitch variability and voice duration were recognized as the prosodic features reliably distinguishing people with ASD from TD individuals.

## Introduction

Autism spectrum disorder (ADS) is a common neurodevelopmental disorder^1^. According to World Health Organization Fact Sheet on June 2021, about one out of 160 children in the world suffers from ASD. This neurodevelopmental disorder is characterized by difficulty in social interaction, atypical patterns of behavior, and communication challenges^2^. In terms of communication difficulties, one of the earliest symptoms of ASD is language impairment^3,4^ that is manifested in different forms, from being completely nonverbal to having unusual prosody^5–7^.

Prosody is concerned with suprasegmental features of speech and refers to the speech rhythm^8^ and also affective, pragmatic and syntactic communicative functions^9^. Therefore, prosody may determine the way that language is perceived by audiences^10^. Considering the fact that cross-linguistic evidence shows the early development of prosodic sensitivity to ambient language in newborns^11,12^, it is important to investigate if prosodic characteristics of the human voice are potent to obtain useful information for neurodevelopmental disorders like ASD.

Acoustic characteristics expressing prosody are pitch (also known as fundamental frequency), duration and intensity^13^ and pitch attributes such as pitch contour, pitch range and pitch variability. The pitch measure is related to the vibrational frequency of vocal cords and pitch contour shows the pitch variability during time evolution and is related to the information content of the utterance^13^. The pitch range is defined as the distance between maximum and minimum pitch values and determines the extent of variation that a speaker uses in a produced utterance. People with ASD show different pitch characteristics as compared with typically developing (TD) individuals^14^. However, inconsistencies are observed between studies. For example, some people with ASD exhibit a wider pitch range, while some other ASD people exhibit a shorter pitch range during speaking compared with TD individuals^14^. Furthermore, some studies showed that people with ASD had higher mean pitch and wider pitch range^15^, while according to other studies, the variation might not be significant^16^ or even different^17^. One possible factor that made the results inconsistent might be the experimental condition that voice is produced^18^. It was shown that emotional state affected acoustic properties of the speech^19^, Furthermore, the mental status during voice production, for example, stress condition, might affect the quality and characteristics of the produced voice^20^.

Duration, the time spent for producing utterances, also seems to be different between ASD and TD groups in a way that people with ASD have longer utterance duration compared with TD individuals^10,13^. This is not in accordance with the other results reported for the duration of the paired syllable^18^ or contrastive argument^17^. Furthermore, the duration for producing stressed and unstressed syllables is more different for TD individuals as compared with people with ASD^16^. Although the utterance duration has been reported to be influenced by the emotional state of TD individuals and is significantly longer in the sad emotional state compared with happy or neutral states, such a difference has not been reported for ASD people^21^.

The intensity of produced voice, sometimes is referred to as voice loudness or voice pressure level (dB), is another measure that has been widely investigated to capture differences between ASD and TD individuals^10,13,22^. Diehl et al. reported that following elicitation of question-like speech, there was no difference between speech intensity of TD and ASD groups^13^. Drimalla et al. found no difference between ASD and TD groups regarding the intensity of produced voice^22^ and Filipe et al. reported that the intensity of voice was not different between ASD and TD groups for both falling and rising intonations^10^. However, according to Olivati et al., the maximum and minimum intensity of vocal productions were significantly different between ASD and TD individuals, in which for both cases, ASD individuals showed higher values for minimum and maximum intensities^23^.

In studies regarding the comparison between prosodic features among ASD and TD individuals, it is important to consider factors such as gender, age, IQ or expressive spoken language of participants. It is well known that speech rate, pitch and voice intensity are correlated factors with speaker age^24^. Also, it was reported that the voice fundamental frequency of children reduced by increasing the age^25^. For school-age typically developing children, the pitch variation was shown to be greater than pre-school samples^26^. Gender is another important factor that affects prosodic features^27^. In general, prosodic attributes of female speakers show higher pitch value, longer duration and a wider pitch range^28^. According to Nadig and Shaw, IQ might also be correlated with acoustic pitch range in high functioning ASD individuals during a structured communication task, while no relationship was found for conversational communication^29^. Furthermore, a correlation between IQ measure and linguistic prosody task accuracy was reported for specific language impairment children^30^. Other factors such as the expressive language of participants or musical background are also effective on the prosodic features of an acoustic utterance. For example, lexical stress assignment is performed in a different manner between different languages^31^. In this regard, several studies used prosodic features for identifying the spoken language of participants^32^. Also, the correlation between prosodic features and factors such as age, gender and IQ is very complex and interaction should be considered between them. As an example for such interaction, it was shown that fundamental frequency changed differently between male and female samples after adulthood (i.e. decrease of voice fundamental frequency in women and increase of fundamental frequency in men); however, such change depends on the age span^33^.

Overall, extensive inconsistencies exist in the literature for the main differences of prosodic features between ASD and TD groups. Performing a systematic review and inclusion of previously conducted studies may help to solve such inconsistencies. By aggregating individual studies, systematic reviews minimize the bias of the obtained results, obtain more reproducible results and increase the power of statistical analyses due to larger sample sizes compared with individual studies. It also generates useful conceptual frameworks and guidelines for future studies by obtaining the effect of eligible confounding factors^34^.

To the best of our knowledge, Fusaroli et al. (2017) have performed the last systematic review about the acoustic features of people with ASD and the differences with TD individuals. According to Fusaroli et al., cumulative results showed that mean pitch and pitch range was the most significant different features between ASD and TD groups (Cohen’s d = 0.4–0.5)^35^. In their study, between-study heterogeneity for the included studies was negligible except for the mean pitch and pitch range. However, due to the lack of sufficient evidence, the reliable pooled effect for ASD for voice intensity and quality of voice was not reported^35^. After the study of Fusaroli et al. several new studies have been performed to investigate the acoustic characteristics of speech articulated by ASD sufferers.

The current study is an update for the last performed systematic review conducted by Fusaroli et al., increasing the study sample size from 30 to 39 and ASD participant sample size from 407 to 910 samples. However, it should be noted that, in Fusaroli et al., some other voice characteristics such as voice quality were also studied. They also considered multivariate studies of acoustic patterns which are out of the scope of the current study. The increased study and participant sample size enabled us to obtain more precise estimates. The main purpose of the current systematic review was to investigate which prosodic features could be considered as reliable markers for discriminating people with ASD from TD individuals. Performing cumulative research by considering newly obtained evidences will increase the sample size and hence improve the statistical power of outcomes. Furthermore, analyses regarding the impact of confounding factors such as the age of participants, gender and the type of voice elicitation tasks on the prosodic characteristics were performed. The result of this study can be used by researchers to develop machine-learning approaches for discriminating ASD and TD individuals or for screening people with ASD. Furthermore, the result can be useful for developing rehabilitation intervention strategies for improving the speaking abilities of people with ASD.

## Results

**Figure 1:**
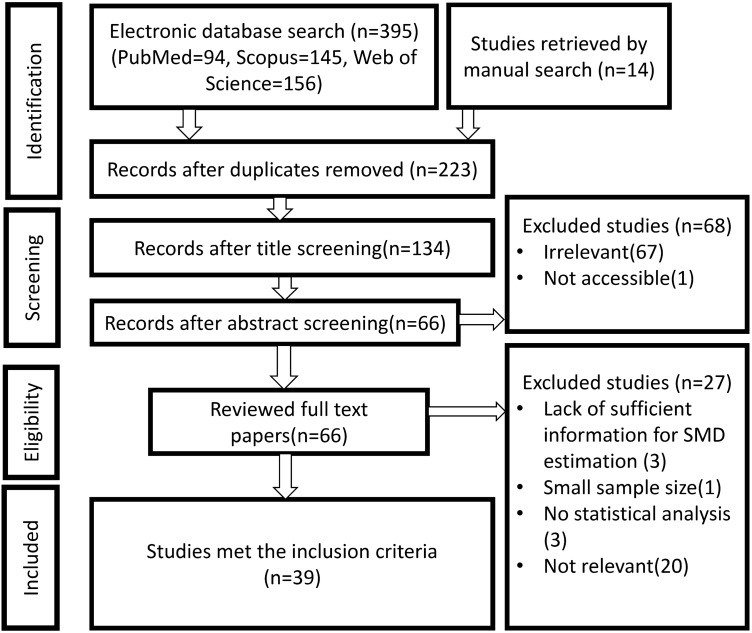
Flow diagram of the search procedure according to the PRISMA guidelines.

The flow diagram for performing the current systematic review according to Preferred Reporting Items for Systematic Reviews and Meta-Analyses (PRISMA) guidelines is shown in Fig. 1.

Initial searches in major databases (PubMed, Scopus and Web of Science) provided 395 studies. Furthermore, by the manual search of the reference list of review articles, related meta-analyses and eligible studies, 14 studies were obtained. After title and abstract screening, 66 studies remained, while only 39 of them contained results that enable us to calculate reliable effect size (i.e. standard mean difference). Although the total sample size of ASD and TD groups were 910 and 850, respectively, separate analysis for each prosodic feature (pitch, intensity, duration and speech rate) was performed with a smaller sample size.

Table 1 shows the retrieved eligible studies that were included in the current systematic review. According to the Newcastle-Ottawa scale (NOS), the quality of all studies was high (NOS ≥ 7) except for Refs.^36–39^ (NOS = 5, 4, 3, 6, respectively). Since the prevalence of ASD is higher in males and ASD is a male-biased disorder^40^, in the case of studies that reported results according to gender, the results for males were only considered. It should be noted that for such studies the sample size for females was relatively small; therefore, the inclusion of effect sizes for the female group increased the risk of small-study effect in the analyses^41^.

**Table 1:** Summary of included studies.

Study (authors, year, ref)	n, n_male_, age		Voice elicitation	Measurements	Outcomes
	ASD	TD			
Arciuli and Bailey (2019)^42^	20, 18, 7.38 ± 1.55	20, 18, 7.21 ± 1.78	Picture-naming strategy	Pairwise variability index (PVI)	Stress contrastivity: ASD < TD
Arciuli et al. (2020)^43^	16, 13, 5.73	16, 3, 4.65	Picture-naming targets	Duration, F0, intensity of the first two vowels for PVI	Results of acoustic analyses indicated no statistically significant group differences in PVIs
Bone et al. (2016)^44^	95, 75, 8.8 ± 2.6	81, 56, 8.3 ± 2.5	Narration	Pitch dynamics, rate of speech, prosodic attributes, and turn-taking	Prosodic variability increases in interactions with higher severity ASD Pitch variability: ASD > TD
**Bonneh et al. (2010**)^45^	41, 32, 5 ± 1.1	42, 17, 5.1 ± 0.7	Picture-naming task	Long-term average spectrum and pitch variability	ASD spectrum was shallower and showed less harmonic structure. pitch range: ASD > TD
**Brisson et al. (2014**)^46^	12, 10, 0.365 ± 0.073	11, 9, 0.309 ± 0.115	Extracted infants’ and mothers’ voice productions recorded through the family home movies	Mean duration and pitch	ASD infants’ productions were not different in duration and pitch, however less complex modulated productions were created by ASDs
Chan and To (2016)^47^	19, 19, 25.72 ± 3.63	19, 19, 25.50 ± 3.21	Recording of narrative production	F0, pitch variability and the total number and the type of sentence-final particles from narrative samples	Pitch range: ASD > TD F0: ASD > TD Pitch variability: ASD > TD
Choi and Lee (2019)^48^	17, NR, 8.23 ± 1.55	34, NR, 8.27 ± 1.725	Conversation samples	Voice intensity variation, prosody, pitch	Intensity, pitch, and intonation change: ASD > TD
**DePape et al. (2012)**^49^	12, 12, 23.2 ± 6.6	6, 6, 26.3 ± 4.0	Verbal responses	Overall range-fall (the difference between the peak and the proceeding lowest pitch value),	High language functioning ASD(HASD) had higher while moderate language functioning had lower pitch range compared with TD, higher range-fall for HASD
Demouy et al. (2011)^50^	12, 10, 9.75 ± 3.5	12, NR, NR	Language assessment tasks	Sentence duration	Sentence duration for all types of Descending, falling, rising and floating sentences: ASD > TD
**Diehl and Paul** (**2013**)^13^	24, 16, 12.31 ± 2.32	22, 15, 12.21 ± 2.64	An instrument designed to assess prosody performance in children	Acoustic measures of prosody	Utterance duration, pitch range, pitch variance and mean pitch: ASD > TD
**Diehl et al. (2009**)^15^	21, 19, 13.58 ± 2.10	21, 19, 13.24 ± 2.09	A cartoon for eliciting narratives and Gestures	Standard deviation in F0, average fundamental frequency across the entire narrative	F0: ASD > TD Pitch variability: ASD > TD
Drimalla et al. (2020)^22^	37, 19, 36.89	43, 21, 33.14	Conversation between the participant and an actress	Prosodic features for each frame: f0, jitter (pitch perturbations), and shimmer (amplitude perturbations) and the root-mean-square energy	F0: ASD > TD
Esposito and Venuti, (2009)^51^	10, 5, 1.4 ± 0.125	10, 5, 1 ± 0.07	Cry Observation codes	Duration	Longer screaming duration for ASD
**Filipe et al. (2014**)^10^	12, 10, 8.58 ± 0.51	17, 10, 8.35 ± 0.49	PEPS-C test for assessing the receptive and expressive prosodic skills of children	Duration, pitch (range, mean, maximum, and minimum), and intensity (mean, maximum, and minimum)	Voice duration , pitch range, mean pitch, maximum pitch: ASD > TD
**Fosnot and Jun** (**1999**)^52^	4, 4, 4–17	4, 4, 4–17	Declarative and question sentences	Mean duration and p range	Longer voice duration in ASD group
**Grossman et al. (2010**)^36^	16, NR, 12.33 ± 2.25	15, NR, 12.58 ± 3.08	Picture-naming task	Intensity and duration of speech	Utterance duration: ASD > TD No statistical difference for intensity was found
Hubbard et al. (2017)^21^	15, 15, 27 (21–42)	15, 15, 21 (18–26)	Evoked elicitation procedure for prosodic production for different emotional context	F0 range and voice intensity	Intensity and F0 range: ASD > TD
**Hubbard and Trauner** (**2007**)^53^	9, 6, 14.5	10, 9, 14.5	Repeat type recorded contents with different intonation	Frequency, amplitude, and duration measurements of recorded speech	ASD exhibited lower pitch peak location accuracy compared with TD Pitch range: ASD > TD
Hudenko et al. (2009)^54^	15, 13, 9.1 ± 0.77	15, 13, 9 ± 0.7	Laugh elicitation	Duration, F0, F0 variability	All acoustic measures were not significant, with the exception of the comparisons between voiced and unvoiced laughter
**Kaland et al. (2013**)^55^	20, 14, 28.9	20, 3, 21.8	Communication task	Pitch analysis	F0 range: ASD < TD
Lehnert-LeHouillier et al. (2020)^14^	12, 3, 12.14 ± 1.84	12, 3, 12.23 ± 1.89	Conversation	Acoustic analysis of a goal-directed conversation task, conversational F0 range	F0 range: ASD > TD
Patel et al. (2020)^18^	55, 45, 16.57 ± 6.62	39, 19, 18.99 ± 5.21	Narration elicitation using a wordless picture book	Mean, range and standard deviation of F0, speech rate, speech rhythm using normalized PVI	F0 variability: ASD > TD
Lyakso et al. (2016)^37^	25, x, 5–14	60, NR, NR	Emotional speech, spontaneous speech, and the repetition of words	Pitch values, max and min values of pitch, pitch range, formants frequency, energy and duration of recorded voice and speech	Pitch values of spontaneous speech: ASD > TD
Nadig and Mulligan (2017)^56^	9, 1, 5.72 ± 1.00	9, 5, 3.065 ± 0.59	Audio stimuli	Mullen scales of early learning for assessing cognitive functioning for receptive and expressive language	ASD and TD groups were not significantly different for repetition accuracy ASD group had higher score for accurate repetition for four syllables
**Nadig and Shaw** (**2012**)^29^	15, 13, 11 ± 0.791	13, 11, 11 ± 2	Conversation task	Pitch range	Pitch range: ASD > TD
Nadig and Shaw (2015)^57^	15, 12, 5.5 ± 1.42	11, 2, 5.66 ± 1.9	Describe a target object	Amplitude, duration and mean pitch	Intensity: ASD < TD Duration: ASD > TD
**Nakai et al. (2014**)^26^	20, 15, 7.9 ± 0.7	21, 10, 7.9 ± 0.1	Picture-naming task	F0 and pitch	Greater pitch variability: ASD > TD
Nayak et al. (2019)^38^	16, 11, 7–18	27, 16, 7–18	General communication	Mean pitch, pitch range, and the standard deviation of pitch	Pitch variability: ASD < TD
Ochi et al. (2019)^58^	62, 62, 26.9 ± 7.0	17, 17, 29.6 ± 7.0	General conversation	log *F*0, intensity, and speech rate; mean and standard deviation for pitch and intensity over the whole session	Standard deviation of intensity: ASD < TD
Olivati et al. (2017)^23^	19, 19, 13.37 ± 6.12	19, 19, NR	Speech-language pathology screening for vocal quality, speech chain, comprehension of simple and complex orders	F0, intensity and duration of recorded voices	Maximum and minimum intensity and distance between maximum and minimum F0 frequencies: ASD > TD Duration: ASD > TD
Paul et al. (2008)^16^	46, 43, 13.2 ± 4.4	20, 17, 7.91–27.42	Constrained production (imitation)	Duration	Stressed syllable duration : ASD < TD
Patel et al. (2020)^18^	55, 45, 16.57 ± 6.62	39, 19, 18.99 ± 5.21	Narration	Mean pitch, speech rate	Speech rate: ASD < TD
**Quigley et al. (2016**)^59^	10, 5, 12.12 ± 0.89	9, 5, 11.95 ± 0.84	Mother–infant social interaction	Mean F0, pitch range and intensity	No significant differences were found between groups
**Scharfstein et al. (2011**)^60^	30, 26, 10.57 ± 1.6	30, 22, 10.60 ± 2	Conversation	Pitch and intensity	Mean vocal intensity: ASD < TD
**Sharda et al.** (**2010**)^61^	15, 14, 6.25 ± 1.5	10, 9 , 7.3 ± 2	Spontaneous speech task	Pitch and pitch range	Pitch, pitch range: ASD > TD
Sheinkopf et al. (2012)^62^	21, 15, 0.5 ± 0.5	18, 8, 0.5 ± 0.5	Audio–video recordings at 6 months of age of participants and Identification of cry episodes	F0 and phonation	F0 for cry: ASD > TD
Unwin et al. (2017)^63^	22, 18, 1	27, 12, 1		F0, Amplitude, first and second formants (F1, F2), Cry duration	Cry duration: ASD < TD
Van Santen et al. (2010)^17^	22, NR, 6.35 ± 1.02	22, NR, 6.57 ± 1.29	Lexical stress task	F0, amplitude and duration	F0: ASD > TD during lexical stress task
Wehrle et al. (2020)^39^	14, 10, 42.5 ± 7.8	14, 11, 37.3 ± 8	Semi-spontaneous speech in the form of task-oriented dialogues	Pitch range, mean F0	ASD group shows more melodic or singsongy intonation style

The bold studies are related to the included studies in the last performed meta-analysis by Fusaroli et al.^35^.

NR shows to not reported values.

The results of this systematic review are as follows.

### Mean pitch value

Twenty-two studies investigated the difference in mean pitch value between ASD and TD individuals. The results of these studies were completely inconsistent. Two studies^14,62^ reported lower mean pitch value for vocal productions of people with ASD, while ten other studies^10,17,22,38,39,47,61,62,64,65^ found higher mean pitch value for ASD individuals. Sheinkopf et al., investigating the acoustic characteristics of infants’ crying sound reported two mean pitch values for pain-related and non-pain-related cries. Furthermore, 11 studies^13,15,18,29,38,45,49,58–60,63^ found no significant difference (p > 0.05) between groups. By analyzing adult male participants (age > 13), Nayak et al. found a higher mean pitch value for ASD than TD group, while for younger male participants (age < 13), the mean pitch value was not different between groups^38^.

**Figure 2:**
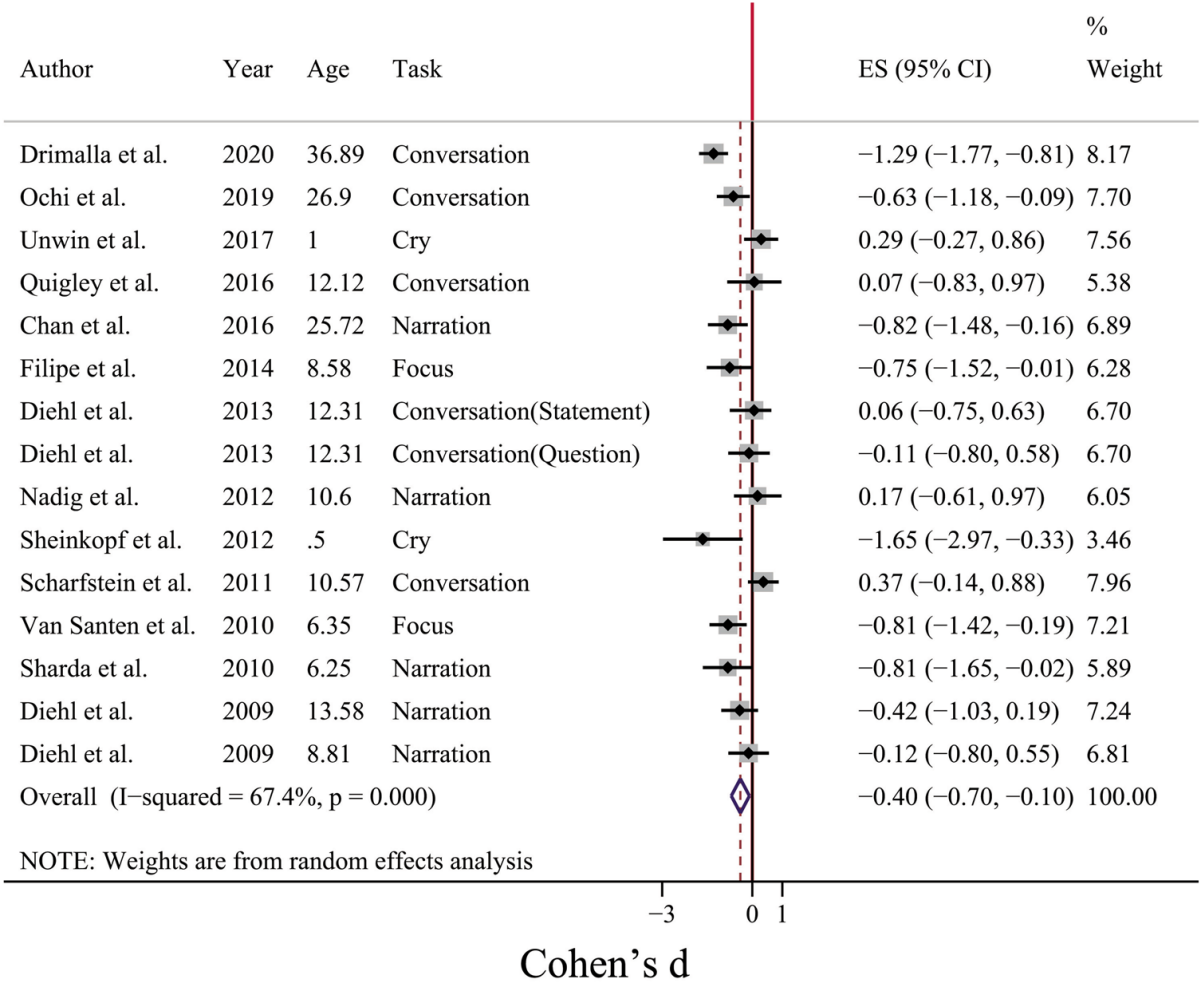
Forest plot for mean pitch value measure. The negative sign shows that the mean pitch value is larger for ASD individuals as compared with TD individuals.

As Fig. 2 represents, the pooled mean difference for included studies (13 studies, 310 people with ASD and 268 TD individuals) was SMD =  − 0.4 (95% CI [− 0.70, − 0.10]), while a moderate to high between-study heterogeneity was observed (I^2^ = 67.4%, p < 0.05). To investigate the source of heterogeneity, two confounding factors (voice elicitation task and the age span of participants) were considered. For this purpose, studies were grouped according to the experimental task that was used for voice elicitation (i.e. Narration, Conversation, Focus and Cry) and the age span of ASD participants (i.e. infancy, childhood, adolescence and adulthood). The results for these subgroup analyses were reported in Table 2.

**Table 2:** Subgroup analyses for mean pitch difference between ASD and TD groups. The elicitation tasks and the age of participants were confounding factors.

	Pooled SMD	Heterogeneity (%)	p-value
**Confounding factor**			
Task type			
Narration	− 0.41 (95% CI [− 0.77, − 0.05])	23.00	0.268
Conversation	− 0.28 (95% CI [− 0.85, 0.29])	80.70	< 0.001
Focus	− 0.79 (95% CI [− 1.26, − 0.05])	0.00	0.915
Cry	− 0.58 (95% CI [− 2.48, 1.31])	71.7	0.029
Age of ASD participants			
Infancy (age ≤ 2)	− 0.58 (95% CI [− 2.48, 1.31])	85.70	0.008
Childhood (age: 2–11)	− 0.30 (95% CI [− 0.76, 0.15])	63.1	0.019
Adolescence (age: 12–18)	− 0.14 (95% CI [− 0.49, 0.21])	0.00	0.718
Adulthood (age > 20)	− 0.94 (95% CI [− 1.36, − 0.52])	40.70	0.185

Student t-test showed that the mean pitch value for TD and ASD groups was statistically different (p = 0.037; t-value = 1.876). For subgroups (according to the type of voice elicitation task and age span), statistical analysis using a two-way analysis of variance (ANOVA) was performed. The ANOVA model of ${y}_{ijt}=\mu +{\alpha }_{i}+{\beta }_{j}+{\gamma }_{ij}+{\varepsilon }_{ijt}$ was used in which alpha showed the effect of different treatments related to the first independent variable (task type, i: Cry, Focus, Narration, Conversation), beta was related to the second independent variable (age, j: Infancy, Childhood, Adolescence, Adulthood) and the gamma coefficient was related to the combination of treatments of two independent variables (i.e. interaction between age and task). The epsilon showed the error term. Multiple comparisons correction using the Bonferroni method was applied to the ANOVA outcome. The results showed no main effect of task type (F(3,8) = 0.36, p = 0.79), age (F(2,8) = 2.07, p = 0.188), and interaction between task type and age span (F(2,8) = 0.29, p = 0.76).

In order to check the impact of elicitation task and age span simultaneously, a more detailed subgroup analysis was performed and the results were reported in supporting materials (see supporting material file, Supplementary Appendix Sect. B).

### Pitch standard deviation

In the current systematic review, in terms of pitch standard deviation, 16 studies reported the difference between ASD and TD groups. The total sample size of the retrieved studies, considered pitch standard deviation, was 305 and 329 samples for ASD and TD groups, respectively. Eight studies^10,13,15,17,47,52,66,67^ reported larger pitch standard deviation for people with ASD, while three studies^26,38,46^ obtained smaller pitch standard deviation for ASD individuals. Other studies reported no significant differences between ASD and TD individuals^26,29,59,60,62,63^. In the study of Nakai et al. lower pitch standard deviation was observed for school-aged ASD individuals, while the pre-school aged group did not show such a difference^26^. Among the included studies, nine studies reported the necessary information for calculating SMD (including 13 effect sizes since some of the studies reported more than one effect size). The pooled mean difference for acoustic pitch standard deviation between ASD and TD groups was SMD =  − 0.07 (95% CI [− 0.55, 0.42], I^2^ = 83.5%, p < 0.01), which was a very small and non-significant difference. Subgroup analyses considering different types of voice elicitation tasks were shown in Table 3. Since just one study was available for adolescence and adulthood, these age spans were excluded for further analysis.

**Table 3:** Subgroup analyses for the difference of pitch standard deviation between ASD and TD groups. The elicitation tasks and the age of participants were confounding factors.

	Pooled SMD	Heterogeneity (%)	p-value
**Confounding factor**			
Task type			
Narration	− 0.14 (95% CI [− 1.14, 0.85])	82.6	< 0.001
Conversation	− 0.16 (95% CI [− 0.75, 0.42])	43.7	0.169
Focus	− 0.11 (95% CI [− 1.11, 0.89])	92.1	< 0.001
Crying	0.56 (95% CI [− 0.68, 1.80])	58.0	0.123
Age of ASD participants			
Infancy (age ≤ 2)	0.21 (95% CI [− 0.54, 0.96])	65.2	0.023
Childhood (age: 2–11)	− 0.05 (95% CI [− 0.87, 0.76])	90.8	< 0.001

The results of this meta-analysis indicated that pitch standard deviation was not significantly different between ASD and TD groups, even when confounding factors were adjusted.

Two-way analysis of variance showed that there was no main effect for task (F(3,6) = 0.4, p = 0.76), age (F(3,6) = 0.92, p = 0.48) or interaction between age and task type factors (F(4,12) = 0.87, p = 0.26).

### Pitch range

Pitch range, the distance between the maximum and minimum pitch values^68^, is one of the measures that along with pitch standard deviation has been used extensively for voice pitch variability assessment^10,21,45,53,61^. In the current study, pitch range was considered as a measure for the broadness of fundamental frequency used by ASD or TD individuals during voice production.

The systematic search found 21 studies for the pitch range difference between ASD and TD groups. Thirteen studies reported the wider pitch range in ASD individuals’ vocal productions^10,13,15,21,29,38,45,52,53,61,62,69,70^, while only one study^55^ reported a narrower pitch range in ASD group. In seven studies, no significant difference between ASD and TD groups was found for pitch range (Refs.^14,18,23,26,39,59^ for one-word and^16^ for pseudowords). Among 21 eligible studies, 14 cases (17 mean difference values) had the necessary information for performing a meta-analysis. The total sample size of these 14 studies was 239 for ASD and 232 for TD individuals. The pooled mean difference for this analysis was SMD =  − 0.78 (95% CI [− 1.34, − 0.21], I^2^ = 89.9%, p < 0.001). As the result showed, there was between-study heterogeneity. To investigate the source of heterogeneity, subgroup analyses were performed considering the age of participants and the voice elicitation method. Table 4 reports the results of the above-mentioned subgroup analyses.

**Table 4:** Subgroup analysis for pitch range difference between ASD and TD groups. The elicitation tasks and the age of participants were confounding factors.

	Pooled SMD	Heterogeneity (%)	p-value
**Confounding factor**			
Task type			
Narration	− 0.58 (95% CI [− 0.94, − 0.22])	91.4	< 0.001
Conversation	− 0.69 (95% CI [− 1.46, 0])	80.7	< 0.001
Focus	− 1.00 (95% CI [− 2.25, 0.24])	57.2	0.097
Cry	No study was found		
Age of ASD participants			
Infancy (age ≤ 2)	No study was found		
Childhood (age: 2–11)	− 1.15 (95% CI [− 2.67, 0.37])	96.4	< 0.001
Adolescence (age: 12–18)	− 0.74 (95% CI [− 1.06, − 0.42])	0.00	0.935
Adulthood (age > 20)	− 0.37 (95% CI [− 1.04, 0.29])	72.6	< 0.001

Student t-test showed that the pitch range value for TD and ASD groups was statistically different (p = 0.002; t-value = 3.21). According to the two-way ANOVA test, there was no main effect for age (F(2,9) = 0.2, p = 0.82), task type (F(2,9) = 0.13, p = 0.88) on standard mean difference of pitch range between ASD and TD groups. Furthermore, the two-way ANOVA test showed that there was no age and task type interaction (F(3,9) = 0.33, p = 0.806).

### Pitch variability

Pitch variability is usually computed according to the standard deviation of fundamental frequencies or the range of fundamental frequencies i.e. the distance between the maximum and minimum pitch values^18^. The pitch standard deviation might better explain pitch variability than pitch range since the latter is a more sensitive measure to outliers. In order to be consistent with the definition of pitch variability in the literature (i.e. considering both pitch standard deviation and pitch range measures), the results of studies of “Pitch standard deviation” and “Pitch range” sections were combined. The systematic search retrieved 22 studies that investigated pitch variability (544 and 561samples for ASD and TD groups, respectively). These studies reported 30 effect sizes. The pooled mean difference for pitch variability measure was SMD =  − 0.462 (95% CI [− 0.84, − 0.08], I^2^ = 88.7%, p < 0.001). This result showed larger pitch variability for ASD group, while between-study heterogeneity was observed. To investigate the source of heterogeneity, subgroup analyses were performed considering the age of participants and the voice elicitation method. Table 5 reports the results of the above-mentioned subgroup analyses.

**Table 5:** Subgroup analyses for the difference of pitch variability between ASD and TD groups. The voice elicitation tasks and the age of participants were confounding factors.

	Pooled SMD	Heterogeneity (%)	p-value
**Confounding factor**			
Task type			
Narration	− 0.41 (95% CI [− 0.81, − 0.01]	53.5	0.154
Conversation	− 0.525 (95% CI [− 1.06, 0.01])	75.5	< 0.001
Focus	− 0.62 (95% CI [− 1.39, 0.16]	94.0	< 0.001
Cry	0.56 (95% CI [− 0.68, 1.80])	58.0	0.123
Age of ASD participants			
Infancy (age ≤ 2)	0.21 (95% CI [− 0.54, 0.96]	65.3	0.021
Childhood (age: 2–11)	− 0.58 (95% CI [− 1.36, 0.19]	94.4	< 0.001
Adolescence (age: 12–18)	− 0.73 (95% CI [− 1.02, − 0.45]	0.0	0.971
Adulthood (age > 20)	− 0.42 (95% CI [− 0.96, 0.13]	68.1	0.008

Student t-test showed that the pitch variability for TD and ASD groups was statistically different (p = 0.008; t-value = 2.53). The two-way ANOVA test showed no main effect for age (F(3,19) = 0.22, p = 0.88), while the main effect for task type (F(3,19) = 8.03, p = 0.04) on the standard mean difference for pitch variability between groups was obtained. The post-hoc analysis showed that for narration-type tasks the pitch variability was larger for ASD samples. Furthermore, analysis of variance showed that there was no interaction between age and task type (F(4,19) = 0.6, p = 0.65).

### Intensity

The difference in the intensity of vocal productions between ASD and TD individuals was found in 12 studies. Two studies^21,23^ reported higher intensity for ASD individuals, while two others^58,60^ found the lower intensity for ASD individuals. In addition, there was not any report of significant differences between ASD and TD groups in terms of voice intensity level in eight studies^10,17,22,29,36,59,62,63^. Among the eligible studies, ten of them had necessary information for calculating the pooled mean difference between ASD and TD groups for acoustic intensity^10,17,21,23,29,58–60,62,63^. Some of them reported more than one mean difference (for minimum or maximum intensity, during different time spans or due to different elicitation methods); therefore, 14 mean difference values were found for performing the meta-analysis. The total sample size for this analysis was 222 people with ASD and 182 TD individuals. The pooled SMD for acoustic intensity deference between ASD and TD groups was SMD =  − 0.14 (95% CI [− 0.58, 0.29], I^2^ = 82.1%, p < 0.001), which indicated the small and non-significant difference between groups (since mean difference contained zero). In Table 6, the results for subgroup analyses, considering the age span of ASD participants and vocal production elicitation methods, were shown.

**Table 6:** Subgroup analyses for voice intensity difference between ASD and TD groups. The elicitation tasks and the age of participants were confounding factors.

	Pooled SMD	Heterogeneity (%)	p-value
**Confounding factor**			
Task type			
Narration	Only one study was available		
Conversation	− 0.07 [− 0.94, 0.8]	90.6	< 0.001
Focus	− 0.24 [− 0.85, 0.38]	57.2	0.097
Cry	− 0.19 [− 0.56, 0.18]	0.0	0.926
Age of ASD participants			
Infancy (age ≤ 2)	− 0.34 [− 0.7, 0.02]	13.7	0.327
Childhood (age: 2–11)	0.29 [− 0.53, 1.1]	85.8	< 0.001
Adolescence (age: 12–18)	Only one study was available		
Adulthood (age > 20)	0.27 [− 0.93, 1.47]	86.4	< 0.001

It should be noted that Ochi et al. (2019) also reported lower variation in the acoustic intensity of people with ASD^58^, while other studies like^60,62^ reported no significant difference between these groups. In the study of Choi and Lee, it was reported that intensity variation for people with ASD was significantly larger (p < 0.05) compared with TD individuals (SMD =  − 0.998, 95% CI [− 1.61, − 0.38])^48^.

Student t-test showed that the voice intensity for TD and ASD groups was not statistically different (p = 0.305; t-value = 0.524). Two-way ANOVA test for standard mean differences for voice intensity between ASD and TD groups showed a main effect of age (F(3,7) = 10.48, p = 0.006), while there were no significant effects for task type (F(3,7) = 0.17, p = 0.911) or the interaction between age and task type (F(4,7) = 0.2, p = 0.356). The post-hoc Bonferroni-corrected contrast analysis showed that the mean value for voice intensity was higher for adolescent ASD subjects in the conversation-type task.

### Speech rate

Patel et al. found a significantly smaller speech rate for people with ASD as compared with TD individuals^18^, while two other studies^29,58^ refused the significant difference between the speech rate of ASD and TD individuals. Sufficient information was available for calculating SMD from three studies including^18,29,58^. The pooled mean difference for the eligible studies (ASD and TD group sample size was 132 and 69, respectively) showed weak and non-significant difference between speech rates of ASD and TD groups (SMD = 0.09 (95% CI [− 0.44, 0.62], I^2^ = 49.4%, p = 0.115)).

### Voice duration

For the difference of mean voice duration between ASD and TD groups, 22 eligible studies were retrieved. The total sample size of the included studies was 257 and 234 for ASD and TD groups, respectively. Ten studies^16,17,29,42,43,46,49,54,58,62^ did not find any statistical difference in voice duration between ASD and TD groups. However, 11 other studies reported longer utterance/word duration for people with ASD^10,13,21,29,36,50,52,58,64,71,72^. Demouy et al. reported four SMD values for different types of intonations (i.e. descending, falling, floating, rising). Another study, which investigated the crying sounds of infants, reported shorter voice duration for ASD children^63^. Among the eligible studies, the standard mean difference could be calculated for 15 studies with 27 mean difference values, since in some studies several mean difference values were reported. Although between-study heterogeneity was observed (I^2^ = 72.1%), the performed meta-analysis for the difference of voice duration between ASD and TD groups obtained the pooled difference of SMD = -0.43 (95% CI [− 0.72, − 0.15], I^2^ = 72.1%, p < 0.01)), which indicated the significant longer duration for vocal productions in people with ASD.

Student t-test showed that the voice duration value for TD and ASD groups was statistically different (p = 0.017; t-value = 2.23). However, the two-way ANOVA test revealed that there was a main effect for age (F(3,20) = 8.68, p = 0.027), while no significant effect was found for task type (F(3,20) = 1.18, p = 0.344) or interaction between age and task type (F(4,20) = 1.37, p = 0.28) on standard mean difference of voice duration between ASD and TD groups. Post-hoc analysis revealed that such difference was mainly due to the statistical difference between childhood (t-value = 1.78, p = 0.048) and adolescence (t-value = 2.09, p = 0.04) subgroups.

**Figure 3:**
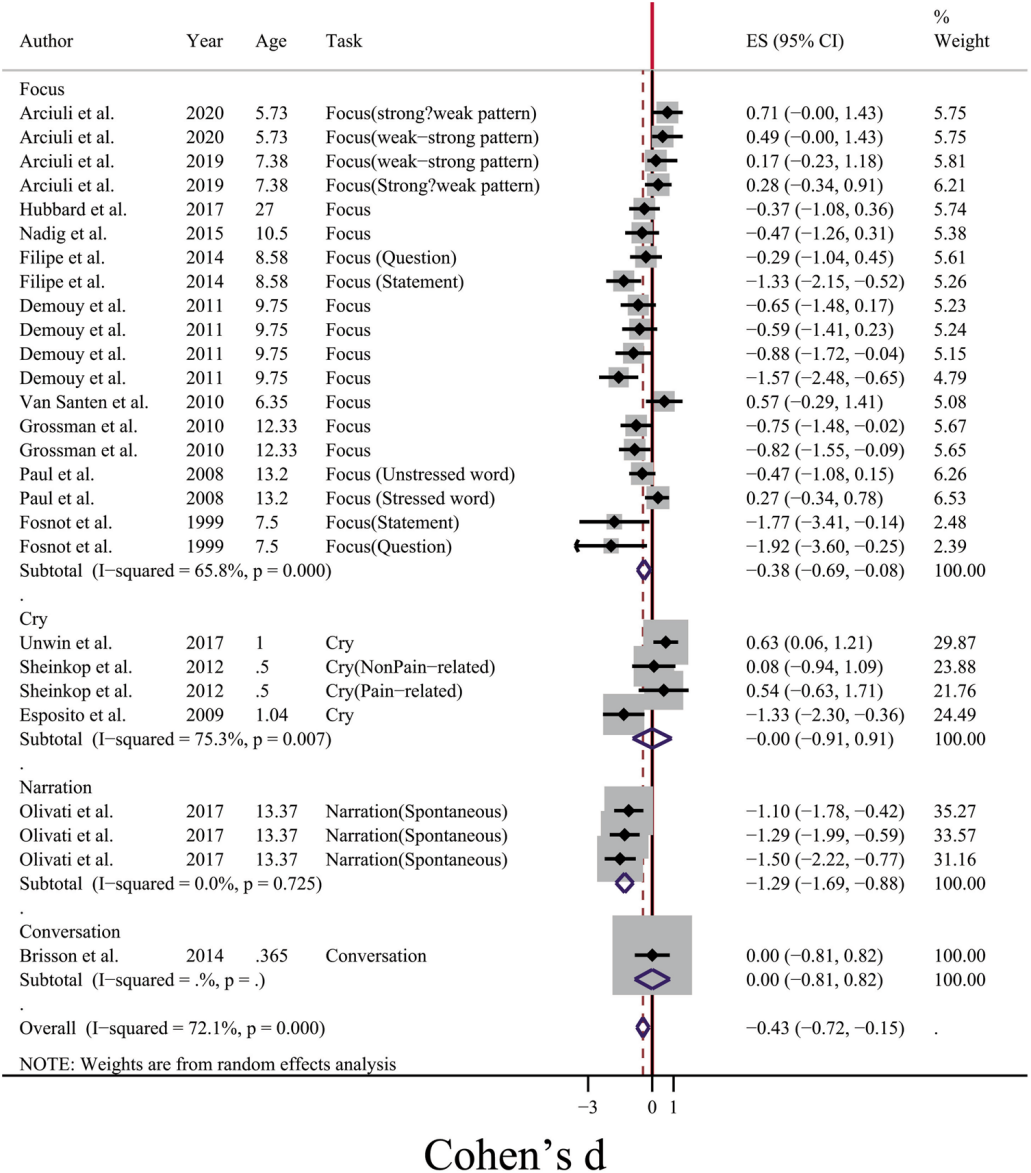
Forest plot for the subgroup meta-analysis of the difference of voice duration between ASD and TD groups. The confounding factor for this analysis was the type of voice elicitation task.

**Figure 4:**
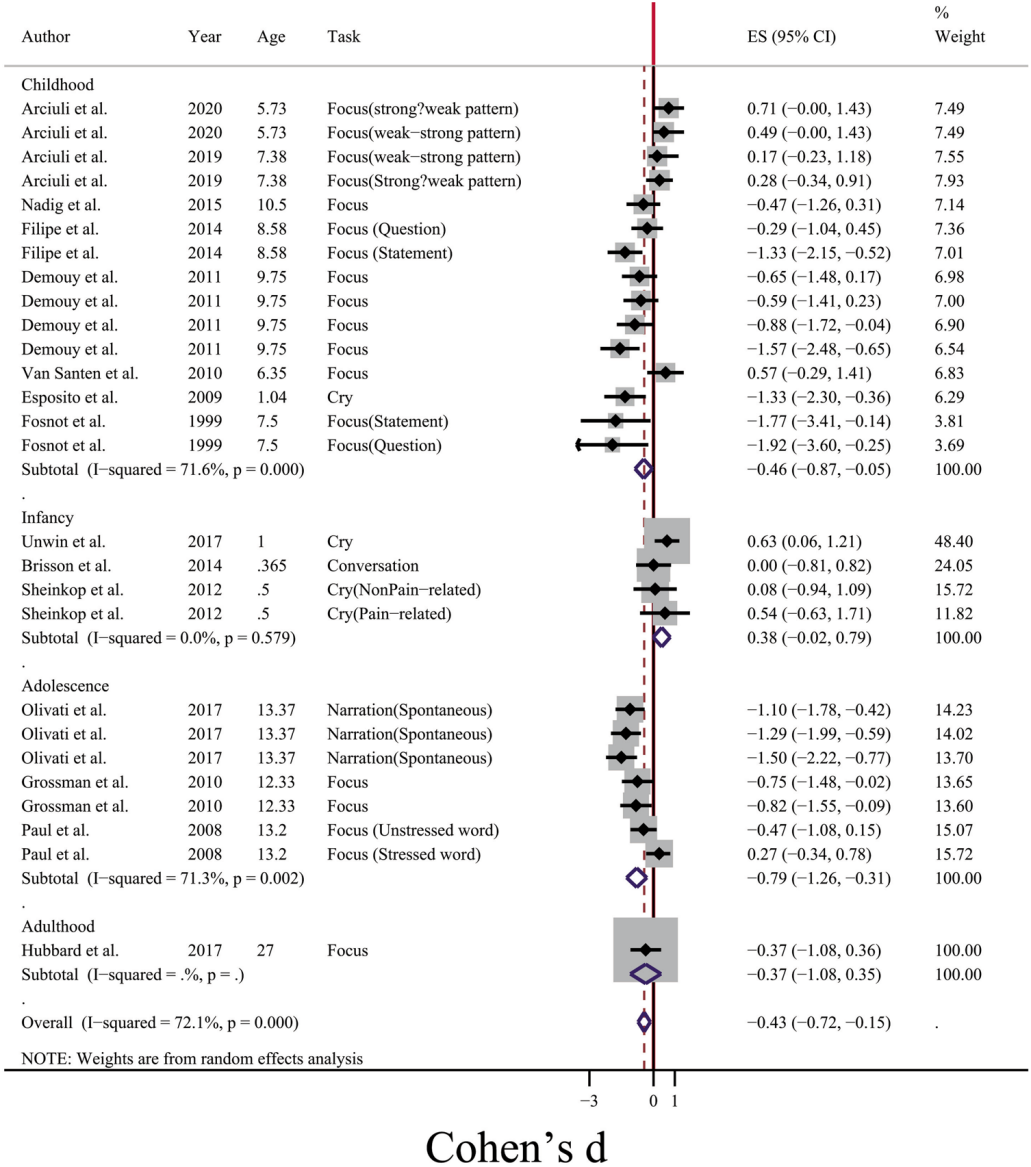
Forest plot for the subgroup meta-analysis of the difference of voice duration between ASD and TD groups. The confounding factor for this analysis was the age span of participants.

From Fig. 3, it was clear that one possible source of between-study heterogeneity for voice duration might be the type of voice elicitation task. When participants were motivated to produce words and sentences in a word repetition or picture naming task (Force category in Fig. 3), between-study heterogeneity was moderate (I^2^ = 65.8%, p < 0.01), while the mean difference of duration was longer for ASD group (SMD =  − 0.38, 95% CI [− 0.69, − 0.08]). For cases in which voice duration was calculated for the crying period, studies were heterogeneous (I^2^ = 75.3%, p = 0.007). In the case of narration, one study^23^ showed non-significant heterogeneity, while due to the common sample population, it could not be considered as a reliable result. Another source of between-study heterogeneity was the age span of participants (see Fig. 4). According to Fig. 4, in the case of vocal production in infants, a non-significant and negligible heterogeneity (I^2^ = 0.00%, p = 0.579) was obtained, and there was a shorter voice duration for crying sounds for people with ASD (SMD = 0.38, 95% CI [− 0.02, 0.79]). For the adolescence and childhood periods, significant between-study heterogeneity was also observed (I^2^ = 71.3% and 71.6%, respectively, p < 0.01).

### Other measures

In some limited numbers of studies, other features such as pitch slope and voice quality were considered. Bone et al. reported that the median pitch slope correlated with ASD severity and a more negatively sloped pitch was observed in children with severe ASD^73^. Pitch slope determines intonation^73^ or the perceived oddness of prosody^74^, where a more negative pitch slope is related to a flatter intonation^73^. The performance of participants during speech production was another measure for comparing ASD and TD individuals^75^. Furthermore, voice quality is used as a measure for comparing ASD and TD individuals; however, there are no universal measures for quantifying the voice quality^35^.

### Publication bias

The results for publication bias according to Egger’s and Begg’s tests were reported in Table 7. The threshold level of 0.05 was used to indicate significant publication bias. The related funnel plot for representing publication bias can be found in Supplementary Appendix D in the Supporting Material.

**Table 7:** Results for assessing publication bias using the Begg’s and Egger’s tests for included studies for different acoustic measures.

Measure	Begg’s test		Egger’s test		
	p value	Z value	p value	Bias	95% CI for bias
Pitch range	0.091	1.69	0.062	− 5.31	[− 10.92, 0.29]
Duration	0.118	1.56	0.053	− 3.24	[− 6.53, 0.046]
Intensity	0.324	0.99	0.144	− 4.95	[− 11.85, 1.94]
Mean pitch	0.928	0.09	0.932	0.17	[− 4.01, 4.34]
Pitch standard deviation	0.583	0.55	0.219	3.19	[− 2.19, 8.57]
Pitch variability	0.668	0.43	0.399	− 1.67	[− 5.66, 2.32]
Speech rate	0.734	0.34	0.653	1.72	[− 12.43, 15.78]

## Discussion

### Study outcomes

Autism spectrum disorder (ASD) is a frequent neurodevelopmental disorder^1^. Recognizing common early symptoms and warning signs of ASD leads to early diagnosis and better treatment assessments. Since ASD is associated with language deficit, in this systematic study, we reviewed the literatures focused on prosodic features of pitch, duration, speech rate and intensity.

As the results of this study (Fig. 2) revealed, the pooled mean difference for mean pitch measure between ASD and TD groups was negative (SMD = − 0.4). The negative sign indicated a larger mean pitch for people with ASD. The confidence interval (95% CI [− 0.7, − 0.1]) did not include zero, therefore, the mean difference should be considered as a significant difference. Subgroup analysis (Table 2) revealed a higher mean pitch for people with ASD especially in adulthood (− 0.94 (95% CI [− 1.36, − 0.52])). This implied that the pitch difference between ASD and TD individuals was specifically significant during adulthood compared to other age spans in which studies showed inconsistent results.

Between-study heterogeneity for mean pitch difference was moderate (I^2^ = 67.4%, p < 0.001). Subgroup analysis revealed that both age and voice elicitation tasks were the sources of heterogeneity (see Table 2). For conversation-type tasks, when social interaction existed during vocal production, between-study heterogeneity was relatively high and significant (I^2^ = 80.7%, p < 0.05). Social interaction problems are the hallmarks of ASD^76^, therefore, a significant difference in the prosodic features of ASD individuals’ vocal production during social interactions in comparison with TD people is not surprising. This might be the reason that conversation-type tasks were an important source of the observed between-study heterogeneity. Furthermore, subgroup analysis according to the age span of participants showed that after childhood period (i.e. in adolescence and adulthood period) the results of studies regarding acoustic mean pitch difference between ASD and TD individuals were more homogenous. Previous studies revealed that mean pitch value decreased during development and it reaches the mature adult voice pitch value in 13–18 years of age^77,78^. Furthermore, Lee et al. reported that the acoustic features converged to a canonical level at the age of 15^79^. The convergence of prosodic features after childhood might be the reason for more homogenous differences for acoustic features in the adulthood age span between ASD and TD groups.

When pitch standard deviation was used, the current meta-analysis did not find a significant difference between ASD and TD groups (SMD =  − 0.07 (95% CI [− 0.55, 0.42]). While for another measure of pitch variability, i.e. pitch range, the current systematic review showed a significantly wider pitch range for the ASD group (SMD =  − 0.78(95% CI [− 1.34, − 0.21]). This can be attributed to the discomfort of ASD patients during speaking^37^. Lyakso et al. reported that a negative/positive trend in pitch variability (falling/rising pitch contour) usually shows the discomfort/comfort state in the speaking, while the flat pitch contour is related to the natural speaking^37^. According to Table 4, pitch range difference reduced during development. Since speech and language abilities enhance during development^80^, people with ASD might feel more comfortable speaking at older ages during social communication and this resulted in the smaller pitch range difference between ASD and TD groups that was observed in our study. It was also in accordance with the findings of Nakai et al. that reported a negative correlation between pitch variation and social reciprocal interaction in Japanese-speaking pre-school children^26^.

The current meta-analysis found a weak mean difference for voice intensity between ASD and TD groups (SMD =  − 0.14 (95% CI [− 0.58, 0.29]); however, the difference was not significant. Subglottic pressure, the rate of airflow, the glottal resistance^81^ and the vocal fold vibration properties^82^ are factors affecting intensity. Kostyuk et al. reported the weakness of respiratory muscles of ASD sufferers and Stewart et al. reported the abnormal lower long airway doublet branching in ASD children^83^ that might influence the air pressure for vibrating vocal cords. Therefore, the difference in voice intensity and pitch properties of vocal productions’ of ASD people can be attributed to the structural abnormalities in vocal tract of the ASD individuals. In addition, the mean pitch is the fundamental frequency (F0) of a voice. The mean value for F0 and its range is mainly determined by the vocal cord characteristic features such as thickness and length^84^. X-ray images from larynx of ASD and their TD counterparts revealed that the hyoid height of people with ASD was lower compared with controls^85^. Different larynx anatomy might be the reason for larger mean pitch and the larger pitch range in ASD individuals.

The pooled voice duration difference between ASD and TD groups showed significantly longer duration for ASD individuals (SMD =  − 0.43 (95% CI [− 0.72, − 0.15]). This result indicated that people with ASD needed more time for producing utterances as compared with TD individuals.

Assessment of publication bias using funnel plot as well as Egger’s and Begg’s rank tests revealed that there was no symptom of publication bias for performed analyses (see Table 7, Fig S3, supporting material). According to Table 7, both Egger’s and Begg’s tests obtained p values larger than 0.05, however, for pitch variability and duration Egger’s test had marginal p values (p < 0.1). This indicated that the result for publication bias obtained by Egger’s test should be considered with caution, especially for pitch variability and voice duration measures.

A deeper investigation in the results obtained by subgroup analyses revealed that the differences of prosodic features between ASD and TD groups were mainly seen for narration-type tasks. For narration-type tasks, significant longer mean pitch value, larger pitch range, higher pitch variability and longer voice duration were observed for people with ASD. While for focus-type tasks, the significant difference between groups was observed for limited numbers of acoustic features (mean pitch and voice duration). For conversation-type tasks, no statistically significant differences were observed (i.e. confidence interval included zero). This useful outcome should be considered for designing future studies.

Considering the age span of participants, pitch range, pitch variability and voice duration were significantly different between ASD and TD groups for the adolescence group. The mean pitch value was also significantly higher for adult ASD participants compared with TD peers. These results showed that the main differences were observed for adolescent and adult age spans. It should be noted that the structural features of the larynx and vocal cords are the most influential factor on the pitch, intensity and variation of a produced voice by human. The majority of structural changes in the larynx and vocal cords begin around puberty, i.e. during adolescence and finally, voice stabilizes in the early years of adulthood. Voice stabilization reduces the intrinsic voice variability^86^ for acoustic features and highlights the inter-subject differences. This might be the reason that why the main differences between acoustic features of ASD and TD groups were mainly seen at older ages (adolescence and adulthood).

### Comparison with the last performed systematic review

The previous systematic review performed by Fusaroli et al. found that the mean pitch and pitch range were two acoustic features that were distinct between ASD and TD groups^35^. According to Fusaroli et al., the standard mean difference for mean pitch was SMD = − 0.41 (95% CI [− 0.68, − 0.15], 16 studies) which is very close to our estimate (SMD =  − 0.4 (95% CI [− 0.7, − 0.1], 22 studies). This confirms that mean pitch value can be considered as a reliable feature to distinguish ASD from TD individuals. For pitch range measure, the previous meta-analysis obtained the pooled mean difference of SMD =  − 0.5 (95% CI [− 0.77, − 0.24], 17 studies), while the current analysis obtained the pooled mean difference of SMD =  − 0.78 (95% CI [− 1.34, − 0.21], 21 studies) that showed the stronger capability of pitch range for distinguishing ASD and TD individuals. In fact, our result obtained a wider pitch range difference between ASD and TD individuals compared with Fusaroli et al.^35^. Inclusion of recently reported studies also showed that voice duration might be another measure that was significantly longer for ASD group (SMD =  − 0.43 (95% CI [− 0.72, − 0.15])). In accordance with the systematic review performed by Fusaroli et al., the current systematic review did not find any evidence for voice intensity, pitch standard deviation and speech rate to be as differentiating features between ASD and TD individuals.

### Study limitations

Even though systematic reviews and meta-analyses provide the framework for combining results of several studies, the obtained results should be taken with caution due to several issues including heterogeneity between studies and publication bias^87^. Furthermore, for the topic of the current study, the spoken language of participants and the task that was used for voice elicitation were different extensively between studies. Such factors were effective on prosodic features^32^ and made studies heterogeneous. Anyway, the outcomes from a systematic review/meta-analysis study can be considered as a starting point in future studies for investigating the effect of potential confounding factors. In this perspective, Fusaroli et al. performed a cumulative yet self-correcting approach according to the outcomes of their previous meta-analysis^35^ in order to propose guidelines for overcoming the naïve shortcoming of a systematic review/meta-analysis study^87^.

In addition, the current study was performed according to the classical method for meta-analysis. However, another choice is Bayesian meta-analysis, which considers that both data and model parameters are random variables. It includes the a priori knowledge in the model and in this way enriches the meta-analysis^88,89^. Finally, during the systematic search, we found several studies that could not be included in the meta-analysis due to missing reported data. The missing data imputation strategies can be effective methods for including such studies in the meta-analysis^90^.

## Conclusion

Several studies have reported altered vocal production in people with ASD. In this regard, it is important to investigate if prosodic characteristics of vocal productions of people with ASD are different enough to be used as the distinguishing factors between ASD and TD individuals. Because children start vocal productions from the first stages of development, reaching this conclusion is promising for ASD sufferers. The result may lead to the early diagnosis of ASD and better outcomes of their assessment. The current systematic review of the studies on prosodic features of vocal productions articulated by ASD sufferers was conducted to find the statistically frequently reported varieties between ASD and TD individuals. This study showed that some features like mean pitch, pitch range, pitch variability and voice duration were discriminative features. However, these findings were dependent on the age span of participants and the type of task used for voice elicitation. For voice elicitation tasks in which interaction with others was required or subjects engaged in a problem-solving task before voice elicitation, discriminative markers obtained lower statistical significance, while prosodic features during general narration showed a statistically significant difference between people with ASD and the normal group. Furthermore, mean differences in discriminative features between ASD and TD groups were usually observed for adolescents and adults. The findings showed that some other prosodic features such as voice intensity, pitch variability or speech rate were not potent to distinguish ASD individuals from TD people. The obtained results can be considered for developing intelligent methods for distinguishing people with ASD from TD individuals. For future works, we propose using enhanced statistical methods such as Bayesian meta-analysis frameworks.

## Materials and methods

For performing the current systematic review, the Preferred Reporting Items for Systematic Reviews and Meta-Analyses guidelines (PRISMA) were used.

### Search procedure

For finding eligible sources in line with the purpose of the current study, three major databases namely PubMed, Web of Science and Scopus were searched using the advanced search engine in each database. For the systematic search, the following search terms were used:(autism OR “Autism spectrum disorder” OR ASD OR “Asperger syndrome”) AND (“phonological disorder” OR “phonological difficulties” OR “phonological impairment” OR “speech disorder” OR “speech impairment” OR “speech difficulties” OR “voice disorder” OR “voice difficulties” OR “voice impairment” OR “phonology disorder” OR “phonology impairment” OR “phonology difficulties” OR phonology OR phonological OR phonetic) AND (“fundamental frequency” OR formants OR “acoustic energy” OR pitch). For searching eligible studies, no restriction on language or date of publication was applied.

### Inclusion and exclusion criteria

As inclusion criteria: (1) Original research articles, conference papers, clinical trial or randomized control trial articles were included. (2) Studies were included if the difference in acoustic properties between autism spectrum disorder and typically developing conditions had been investigated. (3) Only studies were included that had investigated the acoustic features from an articulatory point of view. (4) Studies contained participants with hearing loss or other neurological disorders rather than ASD were excluded.

As exclusion criteria: (1) Review articles or related systematic review studies were excluded, even though their reference lists were searched for finding missing related studies. In addition, case reports and letter to the editor studies were not included. (2) Studies in which ASD group had been compared with people with language impairment and studies on ASD individuals without comparing them with TD control group, were excluded. (3) Studies with a very small sample size (n ≤ 3) were excluded from further analysis. (4) Studies that investigated speech perception or focused on brain mechanisms (for example elicited event-related potentials during speech production) were excluded. 5) Studies that had used specific tests to score verbal abilities or prosodic capabilities of participants^91^ were also excluded.

### Study selection

The search procedure was performed by two independent authors (S.F and E.J) and retrieved references were transferred to a single EndNote library. After duplicate removal and title, abstract and full-text screening, eligible studies were found. Any disagreement in study selection between authors was resolved by discussion. A PICO model (Population: people with autism spectrum disorder and language- or age-matched typically developing group; Intervention: tasks for eliciting voice production in participants, including simple narrative tasks, two-sided interviews, picture-naming tasks and so on; Comparison: intensity, duration and pitch of produced utterance; and Outcome: the standard mean difference between ASD and TD groups) was used to select eligible studies. Some studies were ruled out from further analyses due to their lack of enough information to calculate the standardized mean difference between groups.

### Data extraction

Using a data extraction form, information such as author name, publication year, type of study design, study sample size (number of ASD or TD individuals included in the study), number of male participants in each group, mean age of participants, procedures adopted in the study, the acoustic features used in the study, the main outcomes of the study and calculated or reported mean differences were extracted. For studies reporting several mean differences, all reported differences were considered for further analysis.

In the current study, we focused on acoustic features like intensity (loudness or pressure level), mean and variability of pitch (or fundamental frequency), duration for utterance production and speech rate. Other measures such as turn-taking^44^, correct word repetition rate, voice quality^73^ or pause duration were not considered.

### Between-study heterogeneity, quality assessment and statistical analysis

Between-study heterogeneity was assessed using Cochran's Q-test and I^2^ statistic^92^. I^2^ value higher than 75% was considered as high heterogeneity, lower than 25% as small heterogeneity and between these two edges (i.e. 25% and 75%) was considered as moderate heterogeneity. To assess publication bias, funnel plot as a visualization tool was used and the Begg’s and Egger’s tests were used to quantify the possible bias^93^. Newcastle-Ottawa Scale (NOS)^94^, developed for nonrandomized studies used to evaluate the quality of studies. The difference between ASD and TD groups was calculated based on the standard mean difference (SMD) using Cohen’s d formula. During SMD calculation, the first group was TD and the second group was ASD. In this regard, the negative SMD value implied a larger value for ASD group. The adopted statistical significance level was 0.05. It should be noted that Cohen’s d is   biased upward for small samples. To correct such bias, the corrected d (d*) measure was used using the following formula^95^ during calculation.

1 $${d}^{*}=\frac{{M}_{1}-{M}_{2}}{{SD}_{pooled}}\left(\frac{N-3}{N-2.25}\right)\sqrt{\frac{N-2}{N}.}$$

In which, *M*_*i*_ was the mean value for *i*-th group, *N* was the sample size or number of studies. *SD*_*pooled*_ was the pooled standard deviation and was calculated according to Eq. (2).

2 $${SD}_{pooled}=\sqrt{\frac{({n}_{1}-1){SD}_{1}^{2}+({n}_{2}-1){SD}_{2}^{2}}{{n}_{1}+{n}_{2}-2}}.$$

In Eq. (2), n_i_ was the sample size and *SD*_*i*_ was the standard deviation for *i*-the group.

Statistical comparison between acoustic features among ASD and TD groups was performed according to Kolmogorov–Smirnov normality test followed by independent t-test for normal distributed cases or the Mann–Whitney non-parametric U test for non-normal distribution cases. The significance level of 0.05 was considered for statistical analyses. Analysis of Variance (ANOVA) for testing the differences between groups in terms of two confounding factors (i.e. age span of participants and type of voice elicitation task) was performed (Two-way ANOVA) and post-hoc analysis according to the Bonferroni multiple comparisons correction test was used for finding the possible significant differences. For performing meta-analsis, STATA version 14 (StataCorp, College Station, TX, USA) was used, while for t-test and ANOVA analyses Matlab 2017b(MathWorks, MA, USA) was used.

### Voice production tasks

It is hypothesized that different brain mechanisms are engaged for producing different types of human voices (i.e. unconstrained vs. constrained voice). It was shown that in some neurological and neurodevelopmental diseases the type of voice elicitation task was effective on produced prosodic features^89^. The selected studies were categorized based on different tasks used to elicit vocal production from participants and three categories of constrained voice production, unconstrained voice production and voice produced during crying were considered. In the first category, referred to ‘Focus’, participants were forced to have vocal production in response to a question, request of word imitation, word repetition or picture-naming tasks. Unconstrained category consisted of two subcategories of (1) without interaction with others where vocal production was done during story-telling or general narration tasks and (2) during communication with others. After this, the former was referred to as “Narration” and the latter was referred to as “Conversation”. Considering these two subcategories is important due to the ASD individuals’ impaired social communication behavior^96^.

## Supplementary Information

Supplementary Information.

## Abbreviations

ASD: Autism spectrum disorder
TD: Typically developing
SMD: Standard mean difference
F0: Fundamental frequency
NOS: Newcastle-Ottawa scale
PVI: Pairwise variability index
