# Supplementary material for: Distinctive prosodic features of people with autism spectrum disorder: a systematic review and meta-analysis study
Source: Sci Rep. 2021 Nov 29;11:23093. doi: 10.1038/s41598-021-02487-6 (PMC8630064; doi:10.1038/s41598-021-02487-6)
Supplement: Supplementary file 1 — Supplementary Information. [file 41598_2021_2487_MOESM1_ESM.docx]

**Supporting material for**

**Distinctive prosodic features of people with autism spectrum disorder: A systematic review and meta-analysis study**

Seyedeh Zahra Asghari^1^, Sajjad Farashi^2^*, Saeid Bashirian^3^*, Ensiyeh Jenabi^2^

^1^School of Medicine, Hamadan University of Medical Sciences, Hamadan, Iran

^2^Autism Spectrum Disorders Research Center, Hamadan University of Medical Sciences, Hamadan, Iran

^3^ Department of Public Health, Hamadan University of Medical Sciences, School of Health, Hamadan, Iran

Corresponding Authors: Sajjad Farashi ([sajjad_farashi@yahoo.com](mailto:sajjad_farashi@yahoo.com); ORCID:0000-0002-5082-6391); Saeid Bashirian ([s_bashirian@yahoo.com](mailto:s_bashirian@yahoo.com); ORCID: 0000-0003-2133-087X)

**Appendix A. analysis of prosodic features based on the gender**

ASD is a male-biased disorder and its prevalence is relatively higher among males [^1^](#_ENREF_1). In this regard, the sample population for ASD-related studies contained almost more male samples as compared with female samples. Recently, gender differences in ASD have been the subject of several studies[^2^](#_ENREF_2)^,^[^3^](#_ENREF_3). Parish-Morris et al. (2017) compared male and female people with ASD using pragmatic language markers. They showed that filler words during conversation pause distinguished boys and girls with ASD[^4^](#_ENREF_4). In a narration-type task, Diehl et al. (2009) reported that average pitch standard deviation for high functioning male ASD samples was larger compared with TD matched samples, while for female ASD samples, pitch standard deviation was smaller as compared with TD samples [^5^](#_ENREF_5). Comparison between male and female people with ASD for age span of <13 and >13 years of old showed that the mean pitch value was larger for female samples in both age spans [^6^](#_ENREF_6). Furthermore, the study of Wehrle et al. (2020) during task-oriented dialogues revealed a larger mean pitch and pitch range value for female ASD samples as compared with male ASD samples [^7^](#_ENREF_7).

The majority of researches on prosodic attributes of people with ASD reported the results for groups containing both male and female samples. However, among retrieved eligible studies, we found some limited numbers of studies reported prosodic features for males and females, separately. These studies include [^5^](#_ENREF_5), [^8^](#_ENREF_8), [^6^](#_ENREF_6) and [^7^](#_ENREF_7). Furthermore, for some studies including [^9^](#_ENREF_9), [^10^](#_ENREF_10), [^11^](#_ENREF_11), [^12^](#_ENREF_12), [^13^](#_ENREF_13), [^9^](#_ENREF_9) and [^14^](#_ENREF_14) only male samples were investigated. According to the available data from retrieved studies in the current meta-analysis, the prosodic features of people with ASD considering the gender of participants were investigated and the summary was shown in Table S1.

**Table S1. Studies in which differences between prosodic features were reported according to the gender. SMD means standard mean difference according to corrected Cohen's d (refer to the manuscript, section 5.5). In SMD calculation for ASD group, group 1 was related to females, while for SMD calculation between ASD and TD samples, group 1 was ASD.**

| Study | Task | male/female | Age span | Prosodic feature |  | Male people | Female people | SMD  (95%CI) |
| --- | --- | --- | --- | --- | --- | --- | --- | --- |
| Nayak, 2019 [^6^](#_ENREF_6) | Conversation | 11/5 | Childhood | Mean pitch | ASD | 296.90±47.29 | 311.85 ±48.56 | 0.314  ([-0.749, 1.376]**)** |
|  |  |  |  |  | TD | 297.93±35.11 | 304.74±44.07 |  |
|  |  |  |  |  | SMD  (95%CI) | 0.025  ([-0.811, 0.861]) | -0.153  ([-1.395,1.088]) |  |
|  |  |  | Adolescence | Mean pitch | ASD | 174.33±34.70 | 274.79±42.37 | 2.711  ([1.297, 4.125]) |
|  |  |  |  |  | TD | 156.30±20.65 | 290.85±36.5 |  |
|  |  |  |  |  | SMD  (95%CI) | -0.631  ([-1.488,-0.225]) | 0.406  ([-0.846, 1.658]) |  |
| Wehrle, 2020 [^7^](#_ENREF_7) | Conversation | ASD:10/4,  TD: 11/3 | Adulthood | Mean pitch | ASD | 136±32 | 226±41 | 2.61  ([1.1 4.12]) |
|  |  |  |  |  | TD | 128±32 | 237±50 |  |
|  |  |  |  |  | SMD  (95%CI) | -0.25  ([-1.11, 0.61]) | 0.254  ([-1.257, 1.748]) |  |
|  |  |  |  | Pitch range | ASD | 91±46 | 143±60 | 1.043  ([-0.179,2.265]) |
|  |  |  |  |  | TD | 70±38 | 134±56 |  |
|  |  |  |  |  | SMD  (95%CI) | -0.5  ([-1.37, 0.369]) | -0.154  ([-1.653,1.345]) |  |
| Diehl, 2009 [^5^](#_ENREF_5) | Narration | 19/2 | Adolescence | Mean pitch | ASD | 187.50 ±35.43 | 213.22 ±29.41 | 0.732  ([-0.742, 2.206]) |
|  |  |  |  |  | TD | 169.60±44.25 | 183.74±22.29 |  |
|  |  |  |  |  | SMD  (95%CI) | -0.447  ([-1.09, 0.197]) | -1.13  ([-3.24,0.981]) |  |
|  |  |  |  | Pitch standard deviation | ASD | 50.04 ±10.13 | 45.16 ±6.23 | -0.49  ([-1.954,0.975]) |
|  |  |  |  |  | TD | 41.38±13.12 | 54.08±2.34 |  |
|  |  |  |  |  | SMD  (95%CI) | -0.739  ([-1.396, -0.082]) | 1.869  ([-0.464, 4.255]) |  |

According to Table S1, three studies (including four effects) reported sufficient information for calculating SMD between female (group 1) and male (group 2) subjects with ASD. Also, according to these studies, standard mean difference between female or male TD (group 1) and ASD (group 2) subjects can be calculated. All three studies reported the mean pitch difference between samples. For other features (i.e. pitch range, pitch standard deviation and intensity) only one report was found for each feature. The results for the pooled mean difference for mean pitch values was shown in Fig S1.


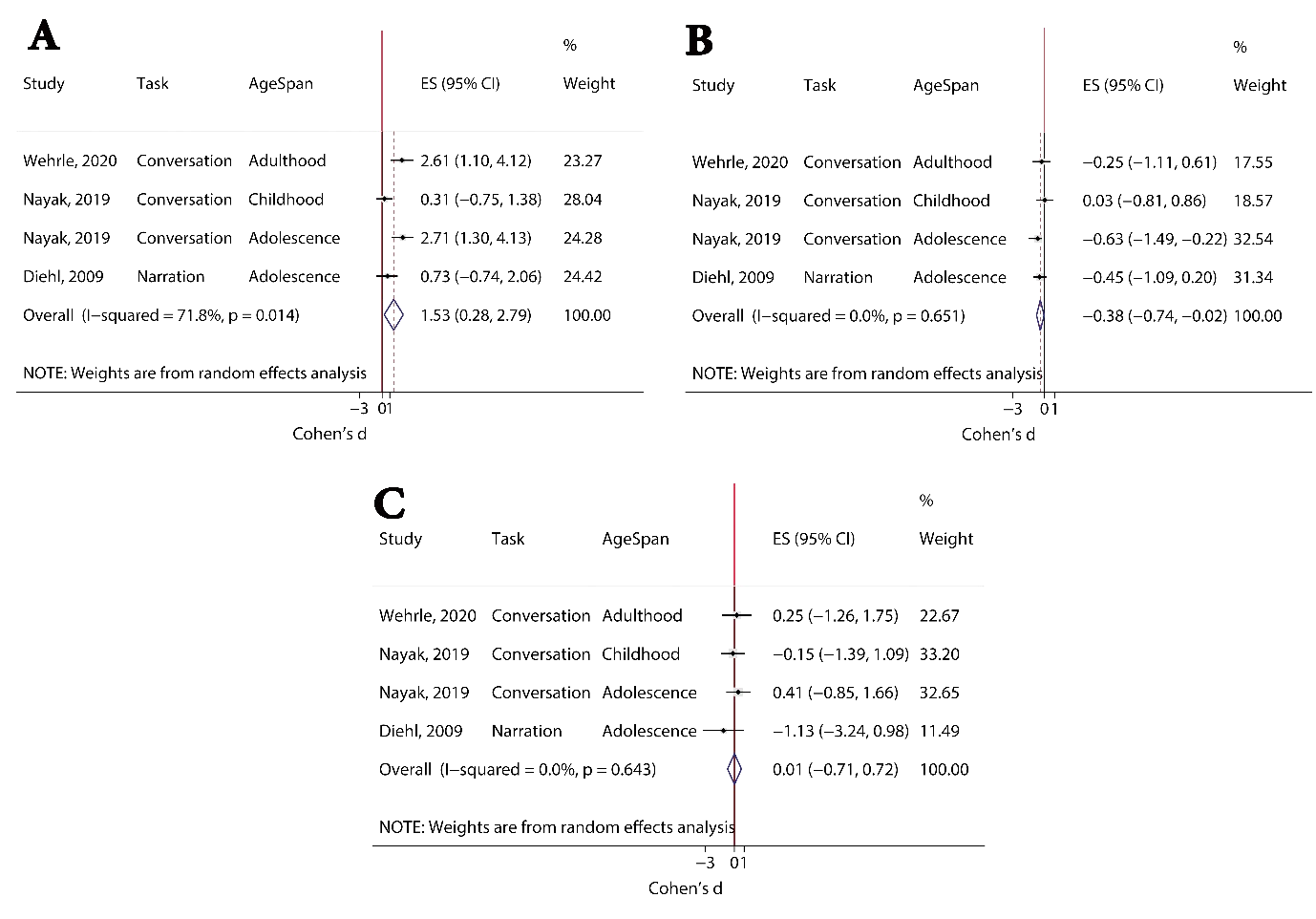


**Fig S1. Forest plot for SMD of mean pitch values between: Fig S1(A): male and female ASD samples, Fig S1(B): male ASD and TD samples, Fig S1(C): female ASD and TD samples.**

According to Fig S1(A), the mean pitch value for ASD female group was significantly larger than ASD male group (SMD=1.53, 95%CI=[0.28, 2.79]). I^2^ measure and Q-test result showed that between-study heterogeneity could not be rejected (I^2^=71.8%, p=0.014). Previous studies reported longer mean pitch values for normal female samples compared with normal males [^15^](#_ENREF_15). This is possibly due to the shorter and thinner vocal cords in females that causes vibration of vocal cords at higher rate (hence higher pitch or voice fundamental frequency value) [^16^](#_ENREF_16). According to the results presented by Fig S1(A), it could be concluded that ASD female samples, like normal female samples, had a larger mean pitch value when compared with male samples.

According to Fig S1(B), male ASD samples showed significantly larger mean pitch value compared with TD peers (SMD= -0.38, 95%CI=[-0.74, -0.02]), while for female samples, the difference between ASD and TD group was not significant (SMD=0.01, 95%CI=[-0.71, 0.72]). There was no obvious heterogeneity between studies for the obtained differences for male and female groups (I^2^=0.0%, p=0.651, and I^2^=0.0%, p=0.643, for male and female groups, respectively). These results showed that the mean pitch value was a more potential marker for distinguishing ASD male samples while it was not suitable for female ASD recognition. Sufficient data was not available to check how age or voice elicitation task might affect the obtained results.

**Appendix B. meta-analysis considering both age span of participants and type of voice elicitation task**

Subgroup analyses reported in Tables 2 to 6 and Fig 3 in the manuscript indicated that the type of voice elicitation task and age span of participants might be effective modifiers and potential sources for observed between-study heterogeneity for differences of prosodic features between ASD and TD samples. It is worth checking if the observed differences were due to the voice elicitation task or the age span of participants. In Tables S2 to S6, subgroup analyses were performed considering the specific effect of voice elicitation tasks in each age span and the specific effect of age of participants on the differences observed for each task. Since the analyses showed no significant difference between voice intensity and speech rate among ASD and TD groups, such features were not considered in the following complementary analyses. In Tables S2 to S6, each reported value indicated the number of included studies (SMD [95%CI], I^2^ statistic, Q-test, p value). For categories with only one study, heterogeneity assessment was not applicable (NA). A dashed line indicated that there was no study for that specific task and age span.

**Table S2. Subgroup analyses for mean pitch difference between ASD and TD groups. Reported values are: number of included studies (SMD [ 95%CI], I^2^ statistic, p value). Significant differences are shown in bold.**

|  | Infancy | Childhood | Adolescence | Adulthood |
| --- | --- | --- | --- | --- |
| Narration | - | 3(-0.24[ -0.78, 0.30], 35, 0.215) | 1(-0.42 [-1.03, 0.19], NA) | 1(-0.82[ -1.48, -0.16], NA) |
| Conversation | - | 1(0.36 [-0.144, 0.877], NA) | 3(-0.004 [-0.43, 0.43] ,0, 0.93) | **2(-0.97 [-1.62, -0.33], 68.1, 0.08)** |
| Focus | - | **2(-0.79[-1.26, -0.05], 0, 0.91)** | - | - |
| Cry | 3(-0.26[ -1.29, 0.76], 71.7, 0.03) | - | - | - |

The following discussion was performed for subcategories with more than one study, since meat-analysis aims to combine the effect sizes reported by more than one study to increase the statistical power of the analysis.

From Table S2, it was obvious that for conversation-type tasks in the adulthood subgroup and focus-type task for the childhood subgroup mean pitch difference was significantly larger for the ASD group (i.e. negative SMD with negative confidence interval limits). For the former, a moderate between-study heterogeneity was observed (I^2^=68.1%, p=0.08), while there was no heterogeneity for the latter case (I^2^=0.00%, p=0.91). According to Table 2 in the manuscript, the polled effect showed a significantly larger mean pitch value for ASD group in narration and focus-type tasks. However, detailed analyses in Table S2 revealed that one study ([^14^](#_ENREF_14)) reported a significant large mean pitch value for the adult subgroup during narration-type tasks. This study might bias the pooled effect size and the confidence interval toward a negative range. For focused-type tasks, only two studies ([^17^](#_ENREF_17)^,^[^18^](#_ENREF_18)) were retrieved which belonged to the childhood subgroup. Based on results from Tables 2 and S2, it could be concluded that the mean pitch of voices produced by children with ASD due to a focus-type task was significantly larger compared with their TD counterparts.

**Table S3. Subgroup analyses for voice duration difference between ASD and TD groups. Reported values are the number of included studies (SMD [ 95%CI], I^2^ statistic, p value). Significant differences are shown in bold.**

|  | Infancy | Childhood | Adolescence | Adulthood |
| --- | --- | --- | --- | --- |
| Narration | - | - | **3(-1.29[-1.69, -0.88], 0, 0.725)** | - |
| Conversation | 1(0.004[-0.82, 0.82], NA)* | - | - | **-** |
| Focus | - | **14 (-0.39[-0.81, 0.00], 71.00, <0.001)** | 4(-0.40[-0.92, 0.11], 60.2,0.056) | 1 (-0.37[-1.08 ,0.35], NA) |
| Cry | **4(0.51[0.05, 0.97], 75.3, 0.007)** | 1(-1.332[-2.301, -0.363], NA) | - | - |

- Analysis of non-crying voices produced by infants

According to subgroup analyses in Table S3, the voice duration in the adolescence subgroup during the narration-type task was significantly longer for ASD group (SMD=-1.289, 95%CI=[-1.694, -0.884]). This result was obtained by including three studies without between-study heterogeneity (I^2^=0.00%, p=0.725). By combining the reported results of four studies, it was shown that the cry duration was shorter for the ASD subgroup during infancy (SMD=0.505, 95%CI=[0.045, 0.966]). Statistical analysis implied the heterogeneity between these studies (I^2^=75.3%, p=0.007).

Comparing Table S3 and Fig 3 in the manuscript, there were three mean difference values (reported by [^9^](#_ENREF_9)) for narration-type tasks performed by the adolescent subgroup. The combination of these mean difference values through a meta-analysis revealed significant longer voice duration for the adolescent ASD subgroup during narration. However, since the samples for these three studies were dependent, any conclusion could be misleading [^19^](#_ENREF_19). For dependent effect sizes, multi-level meta-analysis should be considered [^19^](#_ENREF_19). When the crying duration of infants was considered, TD infants showed significantly longer duration (SMD=0.51, 95%CI=[0.05, 0.97]). Another study ([^20^](#_ENREF_20)) reported a shorter cry duration for children diagnosed with ASD. In addition, Fig 3 in the manuscript showed that people with ASD, during a focus-type task, had longer voice duration compared with TD group (SMD=-0.38, 95%CI=[-0.69,-0.08]). According to Table S3, during a focus-type task, only in the childhood group a longer voice duration was observed, even though studies were heterogeneous (SMD=-0.39, 95%CI=[-0.81, 0.00], I2=71.00, p<0.001).

**Table S4. Subgroup analyses for the difference of pitch standard deviation between ASD and TD groups. Reported values are the number of included studies (SMD [95%CI], I^2^ statistic, p value). Significant differences are shown in bold.**

|  | Infancy | Childhood | Adolescence | Adulthood |
| --- | --- | --- | --- | --- |
| Narration | 1(1.064[0.167,1.96], NA) | - | 1(-0.70[ -1.339, -0.065], NA) | 1(-0.652[ -1.304, 0.000], NA) |
| Conversation | 2(-0.521[-1.171, 0.128], 0, 0.55) | 1(0.231[-0.277,0.739], NA) | - | **-** |
| Focus | - | 5(-0.109[ -1.111, 0.892], 92.1,<0.001) | - | - |
| Cry | 2(0.561[-0.676, 1.798], 58.00, 0.123) | - | - | - |

According to Table S4, for none of the subgroups, significant differences between ASD and TD groups were observed for pitch standard deviation. While for pitch range (Table S5), subgroup analyses showed that adult ASD participants had a significant larger pitch range (SMD=-0.525, 95%CI=[-0.958, -0.093]) for narration-type elicitation tasks as compared with TD group. No obvious between-study heterogeneity was observed for such an outcome (I^2^=0.00%, p=0.844).

**Table S5. Subgroup analyses for pitch range difference between ASD and TD groups. Reported values are the number of included studies (SMD [95%CI], I^2^ statistic, p value). Significant differences are shown in bold.**

|  | Infancy | Childhood | Adolescence | Adulthood |
| --- | --- | --- | --- | --- |
| Narration | - | - | 1(-0.70, [-1.35, -0.04], NA) | **3(-0.53[ -0.96,-0.09], 0, 0.84)** |
| Conversation | - | **2(-1.50 [ -2.13,-0.89], 0.6, 0.32)** | **3(-0.66[-1.18, -0.15], 0, 0.69)** | 1(0.703 [0.06,1.34], NA) |
| Focus | - | 4(-0.98 [-3.12, 1.17], 97.6, <0.001) | **2(-0.85 [-1.36, -0.34],0,0.64)** | 1(-0.85[ -1.36,-0.34], NA) |
| Cry | - | - | - | - |

According to Table S5, for conversation-type tasks, the pitch range was significantly larger for people with ASD during childhood and adolescence (SMD=-1.50, 95%CI=[-2.13, -0.89] and SMD=-0.66, 95%CI=[-1.18, -0.15], respectively). Moderate or small between-study heterogeneity was observed for such outcomes (I^2^=0.6, p=0.32 and I^2^=0.00%, p=0.69, respectively). Furthermore, for focus-type tasks, the ASD adolescent subgroup showed larger pitch range (SMD=-0.85, 95%CI=[-1.36, -0.34]) without significant heterogeneity between studies (I^2^=0.00%, p=0.64). For narration-type tasks adults with ASD exhibited larger pitch range (SMD=-0.525, 95%CI=[-0.96, -0.09]). Comparing Tables 4 and S5, for narration-type tasks, heterogeneity reported by Table 4 was due to combining studies related to adolescent and adult samples since Table S5 showed that for studies focused on adults no significant between-study heterogeneity was observed (I^2^=0.00%, p=0.84). Furthermore, for conversation-type tasks combination of different age spans was the source of reported heterogeneity in Table 4 (I^2^= 80.7%, p<0.001). According to Table 4 in the manuscript, ASD adolescent subgroup exhibited larger pitch range (SMD=-0.74(95%CI=[-1.06, -0.42]), I^2^= 0.00%, p=0.935). This was in agreement with the result of Table S5, wherein adolescents with ASD in all voice elicitation tasks showed longer pitch range as compared with TD group.

**Table S6. Subgroup analyses for difference of pitch variability between ASD and TD groups. Reported values are the number of included studies (SMD [95%CI], I^2^ statistic, p value). Significant differences are shown in bold.**

|  | Infancy | Childhood | Adolescence | Adulthood |
| --- | --- | --- | --- | --- |
| Narration | 1(1.064 [0.167, 1.961], NA) | - | **2(-0.700[-1.156, -0.243],0,0.991)** | **4(-0.564[ -0.924,-0.204], 0,0.932)** |
| Conversation | 2(-0.521[-1.171 ,0.128], 0,0.551) | 3(-0.892[-2.216, 0.433], 89.5,<0.001) | **3(-0.661[ -1.175, -0.148],0,0.668)** | 1(0.703 [0.064,1.342], NA) |
| Focus | - | 9(-0.481[ -1.457, 0.494], 95.5, <0.001) | **2(-0.846[ -1.355, -0.337], 0,0.64)** | 1(-1.372 [-2.452, -0.292], NA) |
| Cry | 2(0.561[-0.676 ,1.798],58.0,0.123) | - | - | - |

When pitch variability was considered (pitch range and pitch standard deviation), ASD adolescence subgroup showed significantly larger pitch variability for narration, conversation and focus-type tasks as compared with TD group. There was no heterogeneity between included studies for different task types. This was in accordance with the results of Table 5 in the manuscript, wherein the pooled effect of included studies revealed larger pitch variability for the adolescent with ASD as compared with TD individuals (SMD=-0.733(95%CI=[-1.017,-0.450]). Also, adult people with ASD exhibited larger pitch variability for narration-type tasks compared with TD individuals (SMD=-0.564, 95%CI=[-0.924,-0.204]). These results were obtained with no heterogeneity between studies (I^2^=0.00%, p>0.05).

The obtained results (Tables S2 to S6) showed that the prosodic features such as mean pitch, pitch range and pitch variability were significantly larger for people with ASD compared with TD individuals during conversation and focus-type tasks. People with ASD show clear deficits during social interactions and communications with others [^21^](#_ENREF_21). Furthermore, previous studies showed that children with ASD had difficulties with verbal problem-solving skills [^22^](#_ENREF_22). Such skills need a conversation with an experimenter and focusing on verbal communication markers. The conversation-type tasks in the current study were related to tasks for producing voices by ASD and TD individuals during communication with an experimenter. In addition, focus-type tasks were related to problem-solving before producing voices. Therefore, it is not surprising if people with ASD exhibit different prosodic features during problem-solving or during communication with others. However, due to the limited number of available studies, it was very difficult to find a causal relationship between age and the differences of prosodic features among ASD and TD groups.

**Appendix C. Relationship between age and acoustic features**

Age is an important factor in the prosodic features of utterances [^23^](#_ENREF_23)^,^[^24^](#_ENREF_24). In the current systematic review and meta-analysis, it was shown that the age span of participant samples should be considered as an important confounding factor. In other words, the standard mean difference between ASD and TD groups might be influenced by age. In Fig S2, considering the available reports (studies) for different prosodic features, the SMD between ASD and TD groups for different age spans (Infancy, Childhood, Adolescence and Adulthood) were depicted.

**
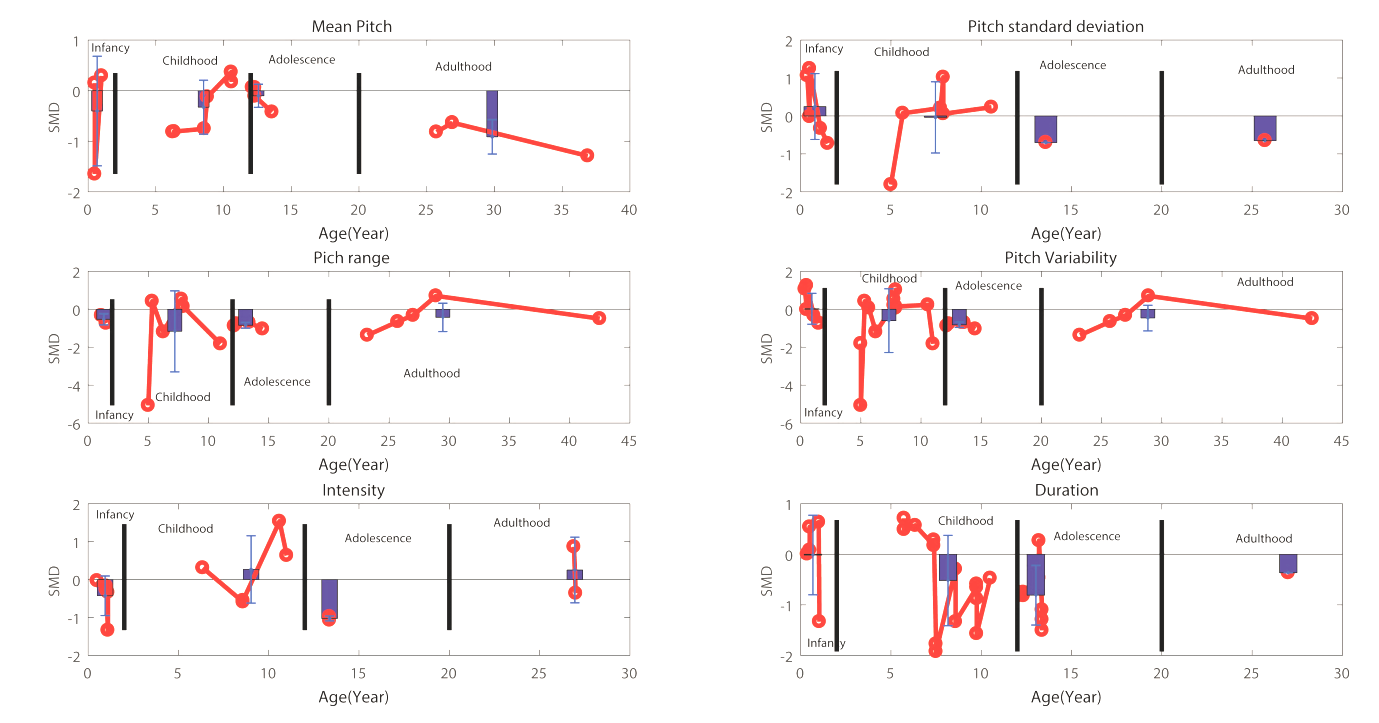
**

**Fig S2. Relationship between the age of participants and the standard mean difference of acoustic features between ASD and TD groups. Vertical lines break the age span into four categories: Infancy, Childhood, Adolescence and Adulthood, respectively. Each bar and errorbar shows the mean and standard deviation of effect size in each category. Line and dot traces are related to the age-SMD relationship for each category.**

From Fig S2, the study sample size for some of the features was not sufficient to discuss the relationship between age and mean difference. However, when features such as mean pitch, pitch standard deviation, pitch range, pitch variability, intensity and duration were considered, it was obvious that a nonlinear relationship was reported between age and SMD during childhood. Such a nonlinear relationship was also seen for the infancy period. For features such as mean pitch, pitch range and pitch variability, a more regular relationship (a cubic or a second-order relationship) was observed for the adolescence and adulthood groups. Due to the limited number of studies included in this study, it is difficult to interpret the causal relationship between age and the difference of prosodic features between ASD and TD groups. Furthermore, some other factors such as gender [^24^](#_ENREF_24), heterogeneity between the design of studies, or even the spoken language by participants [^25^](#_ENREF_25) might be other factors for the observed difference between ASD and TD groups. Since the number of studies included in the current study was limited, it was difficult to find the exact impact of the above-mentioned modifiers on the differences between acoustic features of ASD and TD individuals.

**Appendix D. funnel plots for publication bias analyses**

The funnel plots for visual assessment of publication bias for different prosodic features were shown in Fig S3. Visual inspection showed near-symmetric funnel plots for all features.


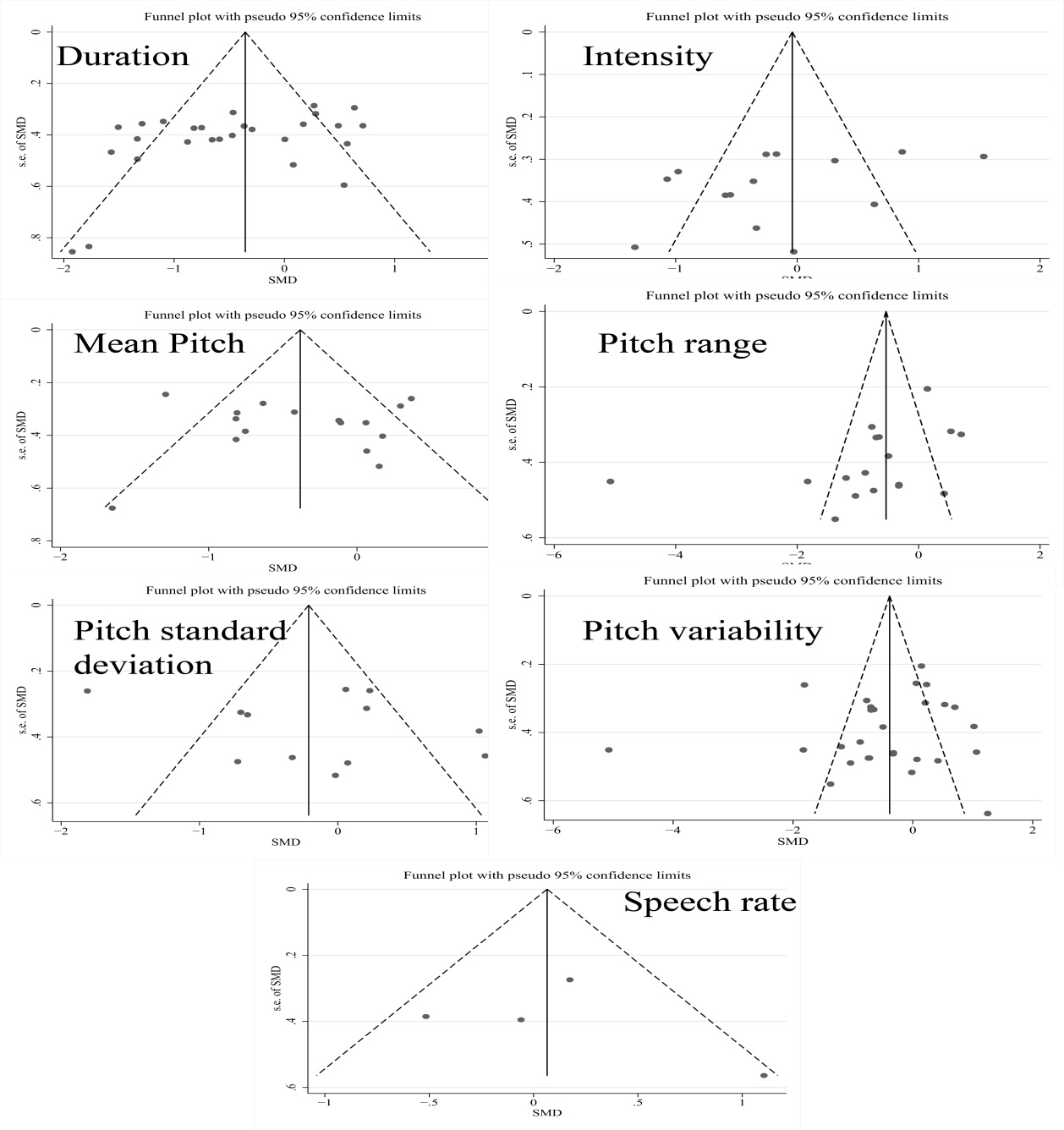


**Fig S3. Funnel plots for representing publication bias for each prosodic feature.**

References

1 Werling, D. M. & Geschwind, D. H. Sex differences in autism spectrum disorders. *Curr Opin Neurol* **26**, 146-153, doi:10.1097/WCO.0b013e32835ee548 (2013).

2 de Giambattista, C., Ventura, P., Trerotoli, P., Margari, F. & Margari, L. Sex Differences in Autism Spectrum Disorder: Focus on High Functioning Children and Adolescents. *Front Psychiatry* **12**, 1063, doi:10.3389/fpsyt.2021.539835 (2021).

3 Zhang, Y. *et al.* Genetic evidence of gender difference in autism spectrum disorder supports the female-protective effect. *Transl Psychiatry* **10**, 4, doi:10.1038/s41398-020-0699-8 (2020).

4 Parish-Morris, J. *et al.* Linguistic camouflage in girls with autism spectrum disorder. *Mol Autism* **8**, 48, doi:10.1186/s13229-017-0164-6 (2017).

5 Diehl, J. J., Watson, D., Bennetto, L., McDonough, J. & Gunlogson, C. An acoustic analysis of prosody in high-functioning autism. *Appl Psycholinguist* **30**, 385-404, doi:10.1017/S0142716409090201 (2009).

6 Nayak, V., Deshmukh, R. & Waghmare, S. Pitch pattern analysis in speech of children with autism spectrum disorder. *International Journal of Innovative Technology and Exploring Engineering* **9**, 4209-4212, doi:10.35940/ijitee.A6119.119119 (2019).

7 Wehrle, S., Cangemi, F., Hanekamp, H., Vogeley, K. & Grice, M. in *Proceedings of the International Conference on Speech Prosody.* 809-813.

8 Nadig, A. & Shaw, H. Acoustic marking of prominence: how do preadolescent speakers with and without high-functioning autism mark contrast in an interactive task? *Lang Cogn Neurosci* **30**, 32-47, doi:10.1080/01690965.2012.753150 (2015).

9 Olivati, A. G., Assumpcao, F. B. & Misquiatti, A. R. N. Acoustic analysis of speech intonation pattern of individuals with Autism Spectrum Disorders. *Codas* **29**, doi:10.1590/2317-1782/20172016081 (2017).

10 DePape, A. M. R., Chen, A., Hall, G. B. C. & Trainor, L. J. Use of prosody and information structure in high functioning adults with Autism in relation to language ability. *Front Psychiatry* **3**, doi:10.3389/fpsyg.2012.00072 (2012).

11 Fosnot, S. M. & Jun, S. in *Proceedings of the 14th international congress of phonetic sciences.* 1925-1928.

12 Hubbard, D. J., Faso, D. J., Assmann, P. F. & Sasson, N. J. Production and perception of emotional prosody by adults with autism spectrum disorder. *Autism Res* **10**, 1991-2001, doi:10.1002/aur.1847 (2017).

13 Ochi, K. *et al.* Quantification of speech and synchrony in the conversation of adults with autism spectrum disorder. *PLoS One* **14**, e0225377, doi:10.1371/journal.pone.0225377 (2019).

14 Chan, K. K. & To, C. K. Do Individuals with High-Functioning Autism Who Speak a Tone Language Show Intonation Deficits? *J Autism Dev Disord* **46**, 1784-1792, doi:10.1007/s10803-016-2709-5 (2016).

15 Schmid, M. & Bradley, E. in *2019 International Congress of Phonetic Sciences.*

16 Biemans, M. The Effect of Biological Gender (Sex) and Social Gender (Gender Identity) on Three Pitch Measures. *Linguistics Netherlands* **15**, 41-52, doi:10.1075/avt.15.06bie (1998).

17 Filipe, M. G., Frota, S., Castro, S. L. & Vicente, S. G. Atypical prosody in Asperger syndrome: perceptual and acoustic measurements. *J Autism Dev Disord* **44**, 1972-1981, doi:10.1007/s10803-014-2073-2 (2014).

18 Van Santen, J., Prud'hommeaux, E. T., Black, L. M. & Mitchell, M. Computational prosodic markers for autism. *Autism* **14**, 215-236, doi:10.1177/1362361309363281 (2010).

19 Cheung, M. W. L. A Guide to Conducting a Meta-Analysis with Non-Independent Effect Sizes. *Neuropsychol Rev* **29**, 387-396, doi:10.1007/s11065-019-09415-6 (2019).

20 Esposito, G., Nakazawa, J., Venuti, P. & Bornstein, M. H. Componential deconstruction of infant distress vocalizations via tree-based models: A study of cry in autism spectrum disorder and typical development. *Res Dev Disabil* **34**, 2717-2724, doi:10.1016/j.ridd.2013.05.036 (2013).

21 Frye, R. E. Social Skills Deficits in Autism Spectrum Disorder: Potential Biological Origins and Progress in Developing Therapeutic Agents. *CNS Drugs* **32**, 713-734, doi:10.1007/s40263-018-0556-y (2018).

22 Alderson-Day, B. Verbal problem-solving difficulties in autism spectrum disorders and atypical language development. *Autism Res* **7**, 720-730, doi:10.1002/aur.1424 (2014).

23 Barnes, D. Age-related changes to the production of linguistic prosody. *Open Access Theses*, doi:<https://docs.lib.purdue.edu/open_access_theses/17> (2013).

24 Torre, P., 3rd & Barlow, J. A. Age-related changes in acoustic characteristics of adult speech. *J Commun. Disord* **42**, 324-333, doi:10.1016/j.jcomdis.2009.03.001 (2009).

25 Gao, Y., Ding, H. & Birkholz, P. An acoustic comparison of German tense and lax vowels produced by German native speakers and Mandarin Chinese learners. *J Acoust Soc Am* **148**, El112, doi:10.1121/10.0001628 (2020).
